# Molecular Analysis of Rising Fluoroquinolone Resistance in Belgian Non-Invasive *Streptococcus pneumoniae* Isolates (1995-2014)

**DOI:** 10.1371/journal.pone.0154816

**Published:** 2016-05-26

**Authors:** Pieter-Jan Ceyssens, Françoise Van Bambeke, Wesley Mattheus, Sophie Bertrand, Frédéric Fux, Eddie Van Bossuyt, Sabrina Damée, Henry-Jean Nyssen, Stéphane De Craeye, Jan Verhaegen, The Belgian Streptococcus pneumoniae Study Group, Paul M. Tulkens, Raymond Vanhoof

**Affiliations:** 1 Unit of Bacterial Diseases, Scientific Institute of Public Health (WIV-ISP), 1050 Brussels, Belgium; 2 Pharmacologie cellulaire et moléculaire, Louvain Drug Research Institute, Université Catholique de Louvain, 1200 Brussels, Belgium; 3 Unit of Foodborne Pathogens, Scientific Institute of Public Health (WIV-ISP), 1050 Brussels, Belgium; 4 Laboratory of Clinical Bacteriology and Mycology, KULeuven, 3000 Leuven, Belgium; Instituto Butantan, BRAZIL

## Abstract

We present the results of a longitudinal surveillance study (1995–2014) on fluoroquinolone resistance (FQ-R) among Belgian non-invasive *Streptococcus pneumoniae* isolates (*n* = 5,602). For many years, the switch to respiratory fluoroquinolones for the treatment of (a)typical pneumonia had no impact on FQ-R levels. However, since 2011 we observed a significant decrease in susceptibility towards ciprofloxacin, ofloxacin and levofloxacin with peaks of 9.0%, 6.6% and 3.1% resistant isolates, respectively. Resistance to moxifloxacin arised sporadically, and remained <1% throughout the entire study period. We observed classical topoisomerase mutations in *gyrA* (*n* = 25), *parC* (*n* = 46) and *parE* (*n* = 3) in varying combinations, arguing against clonal expansion of FQ-R. The impact of recombination with co-habiting commensal streptococci on FQ-R remains marginal (10.4%). Notably, we observed that a rare combination of DNA Gyrase mutations (GyrA_S81L/GyrB_P454S) suffices for high-level moxifloxacin resistance, contrasting current model. Interestingly, 85/422 pneumococcal strains display MIC_CIP_ values which were lowered by at least four dilutions by reserpine, pointing at involvement of efflux pumps in FQ-R. In contrast to susceptible strains, isolates resistant to ciprofloxacin significantly overexpressed the ABC pump PatAB in comparison to reference strain *S*. *pneumoniae* ATCC 49619, but this could only be linked to disruptive terminator mutations in a fraction of these. Conversely, no difference in expression of the Major Facilitator PmrA, unaffected by reserpine, was noted between susceptible and resistant *S*. *pneumoniae* strains. Finally, we observed that four isolates displayed intermediate to high-level ciprofloxacin resistance without any known molecular resistance mechanism. Focusing future molecular studies on these isolates, which are also commonly found in other studies, might greatly assist in the battle against rising pneumococcal drug resistance.

## Introduction

**Fig 1:**
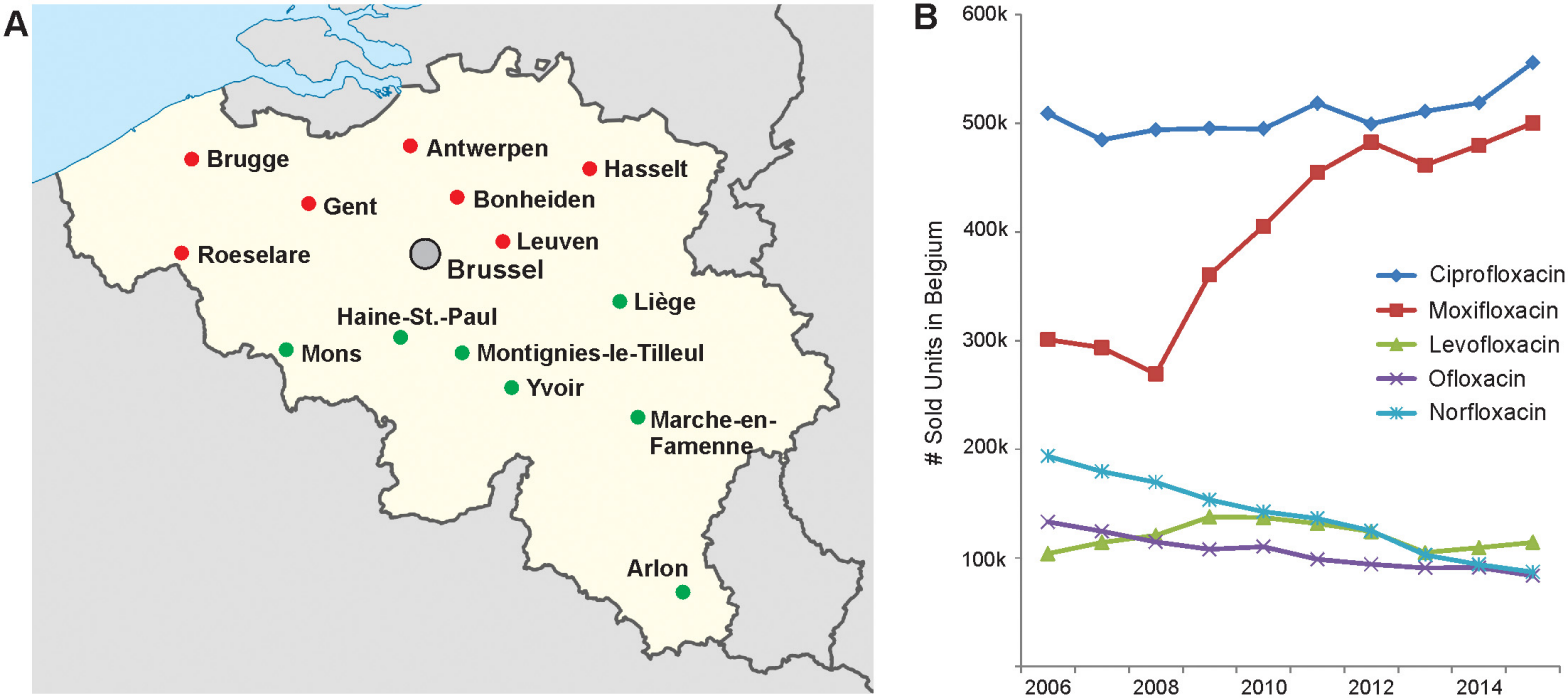
Surveillance of Fluoroquinolone resistance in Belgian non-invasive *S*. *pneumoniae* isolates. A. Clinical laboratories participating in the survey. Participating Flemish and Walloon laboratories are indicated in red and green, respectively. B. Evolution of the total Belgian fluoroquinolone use over the last decade, expressed as yearly sold units of the five main fluoroquinolones (source: IMS dataview, data December 2015).

*Streptococcus pneumoniae* is a major cause of community-acquired respiratory infections including otitis media and pneumonia, as well of serious invasive infections like septicaemia and meningitis [1]. Penicillins and macrolides were mainstay in the treatment of respiratory diseases for decades [2], but the worldwide spread of drug-resistant clones translated into increased usage of fluoroquinolones [3,4]. Fluoroquinolones are synthetic, broad-spectrum antibiotics targeting the DNA gyrase (GyrA/B) and topoisomerase IV (ParC/E) enzymes, which are critically involved in DNA supercoiling and chromosome segregation, respectively [5]. The early fluoroquinolones ciprofloxacin (CIP) and ofloxacin (OFL) target ParC and display poor potency against pneumococci, rapidly leading to emergence of resistance [6]. In the late 1990s, they were replaced by the so-called “respiratory fluoroquinolones levofloxacin (LVX; the active isomer of ofloxacin) and moxifloxacin (MXF) that acts on both enzymes [2]. In Belgium, this has been reflected by steadily declining sales of OFL and norfloxacin while, in contrast, the use of MXF has markedly increased since 2009 (Fig 1) and will probably further expand as its patent has recently expired. Since the global switch to LVX and MXF was established, the worldwide prevalence of fluoroquinolone resistance (FQ-R) in *S*. *pneumoniae* remained below 2% [7] Moreover, it seems unrelated to the serotype switches that were observed upon the introduction of 7- and 13-valent pneumococcal conjugate vaccination [8].

Genetic analyses showed that a first mechanism of FQ-R is through stepwise accumulation of spontaneous mutations in the quinolone resistance determining regions (QRDR) of *gyrA* and *parC*, and rarely *gyrB* and *parE* [9]. The effect of a given mutation depends on the genetic context and the type of fluoroquinolone used [10]. ParC mutations at positions 79 and 83 are most frequently found among pneumococci and are associated with CIP and LVX usage [11]. These first-step mutations lead to a dramatic increase in mutant prevention concentration of all fluoroquinolones [12], enabling prompt selection of secondary and tertiary QRDR mutations in GyrA (mainly positions 81 and 85) required for the FQ-R phenotype [3]. Unlike β-lactam and macrolide resistance mechanisms, QRDR mutations do not appear to be clonally spread and only a minor fraction (0.5–10%) stems from recombination with co-habiting commensal streptococci of the viridans group [13].

In recent years, the role of efflux in low-level *S*. *pneumoniae* FQ-R has become more and more appreciated. Beyond causing a moderate increase in MIC, increased efflux is indeed associated with rising mutational frequencies in the QRDRs [14]. Gene disruption experiments, expression analyses and susceptibility testing in the presence of the efflux pump inhibitors led to current consensus that two distinct transporters, PmrA and PatA/B, are capable of fluoroquinolone efflux [15–19]. The Major Facilitator Superfamily pump PmrA, however, is reported as intrinsically inactive and non-inducible under CIP pressure [20]. More clinical relevance is therefore attributed to the reserpine-sensitive heterogenic ABC efflux pump PatAB. Deletion of this pump in a laboratory strain led to hypersusceptibility to CIP [21], and its expression is induced in the presence of CIP [20]. Moreover, constitutive overexpression of *patA/B* was observed in roughly one-third of clinical isolates with low-level FQ-R [17], and is linked to gene duplication and disruptive mutations in the transcriptional attenuator upstream *patA* [22–24]. Recently, point mutations in PatA were associated with increased CIP resistance by putative enhanced substrate binding [25].

Although most *S*. *pneumoniae* surveillance studies focus on bacteraemia, recent work estimated that for every adult bacteraemic case there are three non-invasive infections [26]. In this paper, we present data on FQ-R in non-invasive pneumococci from a longitudinal surveillance program across Belgian clinical laboratories (1995–2014), spanning the world-wide transit era between the use of early (CIP, OFL) and newer (LVX, MXF) fluoroquinolones. We noted that resistance against the early drugs are markedly on the rise since 2011. By studying the molecular background to dissect the relative contribution of target site mutations versus drug efflux, we identified interesting pneumococcal isolates which confer FQ-R through yet uncharacterized mechanisms.

## Materials and Methods

### Bacterial strains

Non-invasive respiratory clinical isolates of *S*. *pneumoniae* were collected during winter seasons between 1995 and 2014 in 15 clinical laboratories throughout Belgium by members of The Belgian *Streptococcus pneumoniae* Study Group. The access to patient information was encrypted. All isolates were kept at −70°C in Brain Heart Infusion Broth (Difco) containing 10% (v/v) glycerol until transfer to the Scientific Institute of Public Health for susceptibility testing and downstream molecular analyses. The identification of each isolate made by the participating laboratories was confirmed using PCR targeting the autolysin encoding gene *lytA* [26], slide agglutination (Slidex pneumo Kit^™^, BioMérieux, Marcy-l'Étoile, France) and Optochin (Opto-F, bioMérieux) tests, all performed according to the manufacturer’s instructions. For selected strains, capsular sequence typing (CST) was performed by sequence analysis of the *wzh* gene using a dedicated web application (http://www.rivm.nl/mpf/spn/cst/)[27].

### Antibiotic susceptibility testing

For each isolate, the minimal inhibitory concentration (MIC) was determined by broth microdilution as recommended by the US Clinical and Laboratory Standards Institute (CLSI; called National Committee for Clinical Laboratory Standards (NCCLS) at the onset of the study in 1997. The following fluoroquinolones were provided as laboratory standards with known potency by the manufacturers of the original products: levofloxacin and ofloxacin from Aventis Pharma (Mumbai, India), CIP and MXF from Bayer (Leverkusen, Germany). All antibiotics were tested for 16 serial twofold dilutions (0.001–32 μg/mL), with *S*. *pneumoniae* ATCC 49619 [28,29], *S*. *pneumoniae* TPN 881, *Staphylococcus aureus* NCTC 11561 and *S*. *aureus* ATCC 29123 being included as quality control organisms in each series (S1 Table). Interpretation of the results was based on the breakpoints set by the European Committee on Antimicrobial Susceptibility Testing (EUCAST; http://www.eucast.org/). To assess possible synergy between fluoroquinolones and efflux pump inhibitors, commercial E-tests of CIP and MXF (bioMérieux) were applied on MH Blood agar plates containing 0 and 20 μg/mL reserpine. This method was devised after observing that reserpine causes turbidity of broth, preventing a correct reading of the results of the microdilution assay.

### Determination of FQ-R related sequences

The DNA sequences of the QRDRs in *gyrA*, *gyrB*, *parC* and *parE* genes, and of the regulatory regions and coding sequences of *patA* and *patB* were determined by PCR sequencing using the primers listed in S2 Table. All sequences were screened for SNPs in comparison to corresponding regions of FQ-sensitive clinical strains using Clustal Omega (http://www.ebi.ac.uk/Tools/msa/clustalo/); the stability of the *patA* transcriptional attenuator was assessed using the MFold web server (http://unafold.rna.albany.edu/?q=mfold).

### Quantitative real-time PCR

All tested *S*. *pneumoniae* strains were grown overnight in duplicate at 35°C and 5% CO_2_ on Mueller-Hinton agar plates supplemented with 5% defibrinated sheep blood (Bio-Rad Laboratories, Hercules, CA, USA). Bacteria were collected using a sterile loop and suspended in 15 mL Todd-Hewitt Broth medium to an OD_620 nm_ of 0.1–0.2. These samples were incubated at 35°C with occasional stirring to late mid-late exponential phase (OD_620_ ~0.5–0.6), at which point 4 mL of the culture was sampled and cells were harvested by centrifugation (8,000 *x g* for 10 min at 4°C). Cell pellets were rapidly frozen at −80°C until further processing.

Total RNA extraction was performed using the InviTrap^®^ Spin Cell RNA Mini Kit (Stratec Biomedical, Birkenfeld, Germany) according to the manufacturer’s instructions and stored at -80°C. Next, the samples were treated two consecutive times with 2 units TURBO^™^ DNase (Thermo Fisher, Waltham, USA) for 30 min at 37°C, followed by inactivation of the enzyme. To confirm removal of genomic DNA, the *pmrA* gene of *S*. *pneumoniae* was amplified as described elsewhere [19], and RNA concentrations were determined using Qubit fluorescence (Thermo Fisher).

cDNA was synthesized from 150 ng total RNA using the SuperScript^®^ III First-Strand Synthesis System for RT-PCR (Life Technologies) according to the manufacturer’s instructions and using random hexamer primers. Residual RNA was removed using RNase III for 30 minutes at 37°C. Finally, real-time PCR was performed in an iQ cycler (Bio-Rad) in 25 μL reaction mixtures containing 12.5 μL of iQ SYBR Green Supermix (2×), 400 nM of forward and reverse primers and 5 μL of cDNA in RNase/DNase-free water. Primers used for amplification of *pmrA*, *patA* and *patB* are listed (S2 Table), and conditions were used as previously described [19]. Differential gene expression was calculated from the two replicates, as described in Pfaffl et al. [30] and using *rpo*D and *pro*C genes as references to normalize transcript levels, as specified by PrimerDesign (Southampton, UK).

## Results

### Strain collection

A total of 5,602 unduplicated clinical isolates of *S*. *pneumoniae* were included in this study. Isolates were obtained from both ambulatory and hospitalized patients presenting a non-invasive respiratory clinical picture. These strains were collected during the winter seasons in 16 surveys spanning the period 1995 and 2014 by 15 participating clinical laboratories, determinedly selected to obtain a representative sampling of the country (Fig 1). Overall, 47.6% (varying between 40.9% and 53.9%) of the isolates were collected in the Southern part of the country, 44.7% (varying between 39.3% and 49.1%) in the Northern part and 7.6% (varying between 4.7% and 10.1%) in the Brussels area.

### Annual fluoroquinolone resistance rates (1995–2014)

Annual MIC frequency distributions are presented in Table 1. From the onset of our study in 1995, nearly all isolates were classified as non-susceptible to CIP (96.2–100%) and OFL (97.3–100%). Nonetheless, high-level CIP resistance significantly increased from 0% resistant strains in 1995 and 1.4% in 2009, to 9.0% in 2013 (*P* = 0.00025, χ^2^ trend analysis including Bonferroni’s correction) (Table 1). In the same time period, the MIC_50_ of OFL significantly increased from 1 to 2 μg/mL (P < 10^−6^; χ^2^ linear trend analysis, Extended Mantel-Haenszel method), leading to a peak in resistance (6.6%) in 2013. Regarding the respiratory fluoroquinolones, LVX resistance peaked to 3.3% in 2003 and 3.1% in 2012, but remained in general below 2%. Notably, the levofloxacin MIC_50_ also increased significantly from 0.5 to 1 μg/mL since 2012 (P < 10^−6^). MXF was the fluoroquinolone with the highest intrinsic activity on weight basis, with a stable MIC_50_ at 0.06 μg/mL (P = 0.64). Resistance to MXF arose only sporadically, and remained <1% throughout the entire study period (Table 1).

**Table 1:** Yearly percentage of isolates displaying indicated MIC (μg/mL) against four fluoroquinolones. The MIC_50_ values are indicated with an asterisk. The breakpoints (separating the isolates according to their susceptibility to each drug) are those set by EUCAST.

**CIPROFLOXACIN**		**Susceptible**				**Intermediate**				**Resistant**				
**Year**	**# strains**	**0.015**	**0.03**	**0.06**	**0.12**	**0.25**	**0.5**	**1**	**2**	**4**	**8**	**16**	**32**	**% res.**
1995	143	-	-	0.7	2.8	18.9	34.3*	35.7	7.7	-	-	-	-	**0.0**
1997	162	-	-	-	-	6.8	17.9	61.7*	11.7	2.5	0.6	-	-	**3.1**
1999	227	-	-	0.4	0.4	6.2	30.8	47.1*	13.2	1.8	-	-	-	**1.8**
2001	334	-	-	-	0.9	12.9	38*	38	6.6	3	0.6	-	-	**3.6**
2003	391	-	-	0.5	3.1	11.3	25.1	46.3*	9.5	2.6	1.8	-	-	**4.4**
2004	424	-	0.2	1.2	1.9	14.2	37.3*	36.3	6.6	2.1	-	0.2	-	**2.3**
2005	447	-	0.2	1.1	2.5	12.8	35.6*	40.5	6	0.9	0.2	0.2	-	**1.3**
2006	430	-	-	0.2	1.4	7.4	28.6	53.7*	8.1	0.5	-	-	-	**0.5**
2007	413	-	-	0.2	1.5	7.7	30	56.7*	1.7	1.5	0.2	0.5	-	**2.2**
2008	448	-	-	0.2	0.4	4.7	16.1	73.4*	4.7	-	-	0.4	-	**0.4**
2009	413	-	-	-	1.9	6.5	44.1*	44.1	1.9	1	0.2	0.2	-	**1.4**
2010	370	-	-	0.8	2.7	10.8	26.2	55.1*	1.9	2.2	-	-	0.3	**2.5**
2011	368	-	-	0.3	0.5	4.6	14.9	46.2*	29.6	2.2	1.1	0.5	-	**3.8**
2012	351	-	-	-	0.3	1.1	14.2	46.4*	29.9	7.1	0.6	0.3	-	**8.0**
2013	369	-	-	-	-	3	12.5	38.8*	36.9	7.3	1.1	0.3	0.3	**9.0**
2014	312	-	-	-	-	0.6	9.9	49.7*	33	6.4	-	0.3	-	**6.7**
**OFLOXACIN**		**Susceptible**			**Intermediate**					**Resistant**				
**Year**	**# strains**	0.03	0.06	0.12	0.25	0.5	1	2	4	8	16	32	64	**% res.**
1995	143	-	-	-	5.6	22.4	48.9*	15.4	7.7	-	-	-	-	**0.0**
1997	162	-	-	-	-	7.4	43.8*	32.1	15.4	1.2	-	-	-	**1.2**
1999	227	-	-	0.4	0.8	10.6	45.8*	27.3	13.2	1.8	-	-	-	**1.8**
2001	334	-	-	-	2.1	11.4	46.1*	30.2	6.6	3.3	0.3	-	-	**3.6**
2003	391	-	-	0.5	2.8	13.6	40.4*	29.2	9.2	3.1	0.8	0.5	-	**4.4**
2004	424	-	-	1.1	6.8	13	58*	12	6.6	2.1	0.2	-	-	**2.3**
2005	447	-	-	2.7	3.6	25.7	44.5*	16.1	6	1.1	0.2	-	-	**1.3**
2006	430	-	-	0.5	1.8	11.6	51.6*	26	7.9	0.5	-	-	-	**0.5**
2007	413	-	0.5	-	1.5	9.7	51.1*	33.7	1.7	1.3	0.7	-	-	**2.0**
2008	448	-	-	0.2	0.9	4	56*	33.7	4.7	-	0.4	-	-	**0.4**
2009	413	-	-	-	1.9	9	46.7*	39	2.4	0.5	0.5	-	-	**1.0**
2010	370	-	-	1.1	3.5	9.2	48.9*	33	2.7	1.4	0.3	-	-	**1.7**
2011	368	-	0.3	-	1.4	4.3	32.1	50.5*	9.2	1.6	0.5	-	-	**2.1**
2012	351	-	-	-	0.9	2.3	32.8	51.6*	11.1	1.4	-	-	-	**1.4**
2013	369	-	-	-	0.3	4.6	31.7	48.5*	8.4	6	0.3	0.3	-	**6.6**
2014	312	-	-	-	-	1	27.6	64.1*	4.5	2.6	-	0.3	-	**2.9**
**LEVOFLOXACIN**		**Susceptible**							**Resistant**					
**Year**	**# strains**	**0.03**	**0.06**	**0.12**	**0.25**	**0.5**	**1**	**2**	**4**	**8**	**16**	**32**	**64**	**% res.**
1995	143	-	1.4	2.8	19.6	40.6*	30.1	5.6	-	-	-	-	-	**0.0**
1997	162	-	-	0.6	8	58.6*	26.5	4.9	1.2	-	-	-	-	**1.2**
1999	227	-	0.4	-	2.6	37.9	44.1*	13.2	1.8	-	-	-	-	**1.8**
2001	334	-	-	1.2	9	47.6*	33.2	6.3	2.4	0.3	-	-	-	**2.7**
2003	391	-	0.5	3.6	13.6	31.7*	41.4	5.9	1.8	1.5	-	-	-	**3.3**
2004	424	0.5	0.7	3.8	14.2	42.7*	30.2	5.2	2.6	-	0.2	-	-	**2.7**
2005	447	0.9	2	4.5	22.6	48.1*	15.9	5.4	0.4	-	0.2	-	-	**0.6**
2006	430	0.2	1.2	2.1	9.3	28.6*	53.7	8.1	0.5	-	-	-	-	**0.5**
2007	413	0.2	0.5	2.2	13.8	58.1*	23.5	0.7	0.2	0.7	-	-	-	**0.9**
2008	448	0.2	-	1.1	6.9	60.7*	26.1	4.2	0.2	-	0.4	-	-	**0.6**
2009	413	-	1.2	5.3	30.8	46.2*	15	0.7	0.2	0.5	-	-	-	**0.7**
2010	370	0.3	3.5	4.3	17	55.9*	15.7	2.4	0.5	0.3	-	-	-	**0.8**
2011	368	0.3	0.5	3	10.1	37*	41.3	6.8	0.5	0.5	-	-	-	**1.0**
2012	351	-	-	0.9	3.7	41.3	39*	12	2.8	0.3	-	-	-	**3.1**
2013	369	-	-	1.4	2.7	35	49.3*	10.3	0.8	0.3	0.3	-	-	**1.4**
2014	312	-	-	0.6	2.2	30.8	59.6*	6.1	0.3	-	0.3	-	-	**0.6**
**MOXIFLOXACIN**		**Susceptible**							**Resistant**					
**Year**	**# strains**	**0.008**	**0.015**	**0.03**	**0.06**	**0.12**	**0.25**	**0.5**	**1**	**2**	**4**	**8**	**16**	**% res.**
1995	143	-	9.1	33.6	38.5*	13.3	0.7	-	-	-	-	-	-	**0.0**
1997	162	-	0.6	12.3	38.9*	44.4	3.1	0.6	-	-	-	-	-	**0.0**
1999	227	0.4	1.8	11	40.1*	30.4	12.8	2.6	0.9	-	-	-	-	**0.9**
2001	334	0.6	6.3	9.3	43.7*	32.3	5.4	1.5	0.6	0.3	-	-	-	**0.9**
2003	391	1	6.6	13.6	30.2*	36.8	10.5	0.8	-	0.3	0.3	-	-	**0.6**
2004	424	0.5	4.5	17	39.4*	30.2	8	0.2	-	0.2	-	-	-	**0.2**
2005	447	1.1	4	18.6	39.6*	28.2	6.9	1.3	-	0.2	-	-	-	**0.2**
2006	430	1.8	4.7	17	41.4*	30.9	-	0.2	-	-	-	-	-	**0.0**
2007	413	0.7	2.9	11.1	43.1*	30	11.4	-	0.5	0.2	-	-	-	**0.7**
2008	448	0.2	0.9	7.4	38.6*	46.4	6.9	-	-	0.2	-	0.2	-	**0.4**
2009	413	0.2	5.3	11.1	51.3*	25.2	6.3	0.2	0.2	-	-	-	-	**0.2**
2010	370	-	5.4	11.6	49.5*	26.8	5.7	0.8	-	-	0.3	-	-	**0.3**
2011	368	0.3	3.3	12.8	48.9*	27.2	6.5	0.5	0.5	-	-	-	-	**0.5**
2012	351	-	2.3	5.4	48.1*	36.5	6.6	0.9	0.3	-	-	-	-	**0.3**
2013	369	-	1.4	9.5	53.9*	30.1	4.3	-	-	0.8	-	-	-	**0.8**
2014	312	-	0.3	8.3	52.6*	34.3	4.2	-	-	0.3	-	-	-	**0.3**

**Fig 2:**
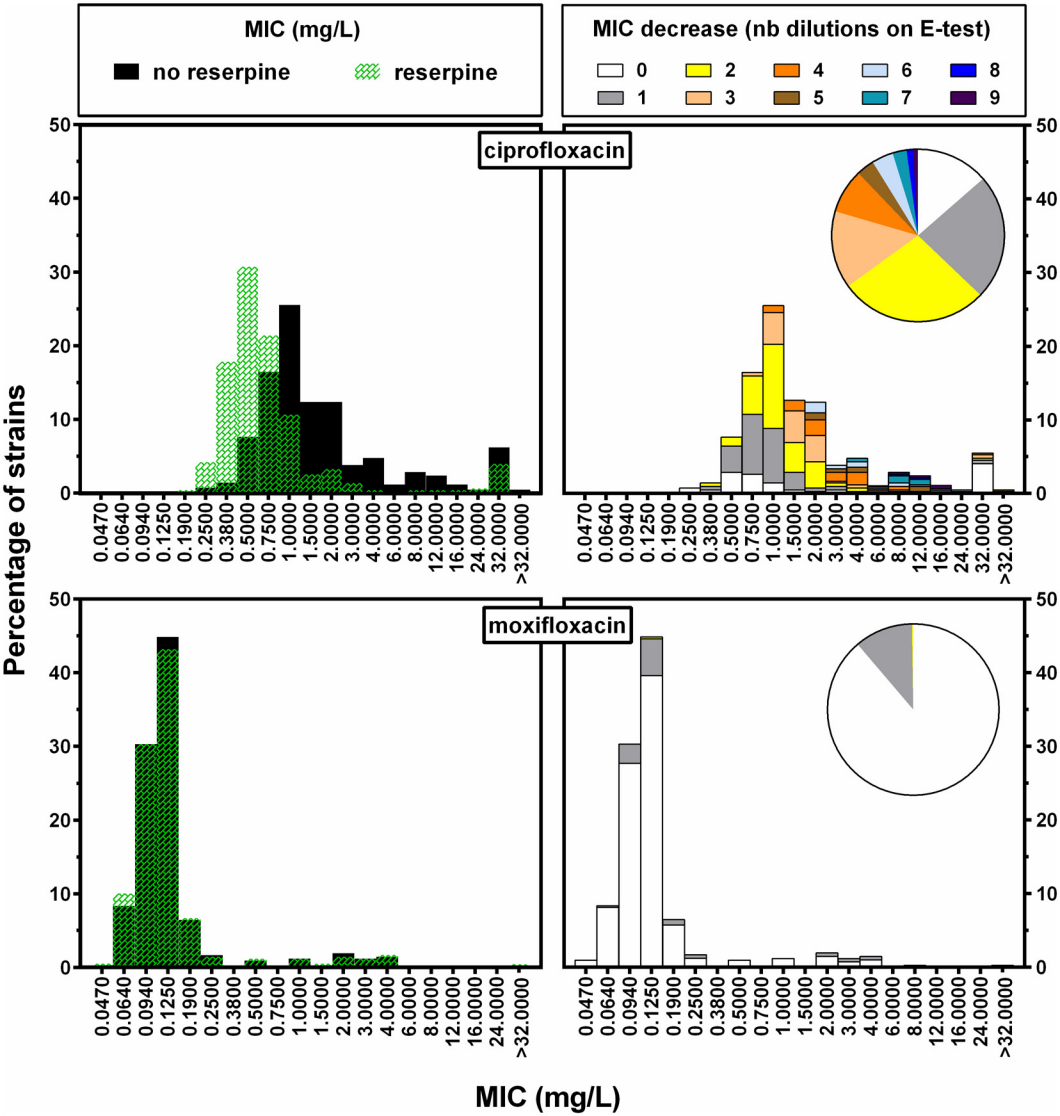
MIC distributions of ciprofloxacin and moxifloxacin (E-test method) for 422 non-invasive *S*. *pneumoniae* isolates collected in Belgium between 1995 and 2014. Left-hand panels: MIC distributions determined in the absence (control; black) or presence (green) of 20 mg/L reserpine. Right-hand panels: reduction of MIC (in blocks of 0.5 log_2_ dilutions from 0 to 3 log_2_ dilutions) after addition of 20 mg/L reserpine and plotted as a function of the MIC distribution of the isolates in the absence of reserpine.

Next, we investigated the influence of the role of efflux in fluoroquinolone resistance using the efflux pump inhibitor reserpine. Hereto, we selected 422 pneumococcal isolates displaying varying MIC_CIP_ and repeated the MIC testing of CIP and MXF in the presence of reserpine (MIC_CIP/MXF+R_). We observed that for 85 (20.1%) isolates, at least a fourfold decrease in MIC_CIP_ was achieved upon addition of the efflux pump inhibitor (Fig 2), which is a common threshold for the definition of an efflux phenotype [31,32]. For 57 (13.5%) isolates there was no effect, while in 16 cases (3.7%) this reduction was very drastic and caused a decrease to up to nine MIC_CIP_ dilutions, accounting for the entire resistance phenotype. In contrast, MXF MICs were much less decreased by the addition of reserpine as the maximal effect was a two-fold reduction in 45 (10.7%) strains (Fig 2). Of note, we used E-tests for all analyses which included reserpine, and recorded MICs which were generally higher than with the corresponding microdilution method: 52.8% one or less, and 90.2% two or less dilutions difference in CIP MICs, and 56.7% one or less, and 89.2% two or less dilutions difference for MXF (S4 Table). Comparable deviations have been reported elsewhere for other Gram-positive bacteria [33–35], and could be attributed to a conservative interpretation due to insufficient growth of the bacterial lawn.

### Analysis of QRDR regions and serotypes

For the same set of 422 isolates, the QRDR of all *gyrA*, *gyrB*, *parC* and *parE* genes were sequenced (Table 2 and S4 Table). To dissect the influence of QRDR from efflux-mediated resistance, various genotypes were grouped according to the MIC of CIP when tested in the presence of reserpine (MIC_CIP+R_) for each strain. Firstly, this allowed identifying a magnitude of topoisomerase mutations unrelated to FQ-R, most prevalent being ParC K137N, K57T and ParE I460V [8–10] occurring in 14.7, 2.2 and 81.0% pneumococcal isolates with MIC_CIP+R_ <1 μg/mL, respectively. Secondly, we identified signatures of recombination with members of the *S*. *mitis* group, judged by the presence of ParC S52G, N91D and/or GyrA S114G substitutions [13], in 10.4% of the strains. These recombinant genes were already identified at the onset of our study, but no significant increase in topoisomerase recombination was noted by 2014.

**Table 2:** Overview of the various FQ-R genotypes encountered in 422 clinical *S*. *pneumococci* strains. Signature residues of the viridans group of streptococci [13] are indicated in bold.

MIC_CIP+R_ (μg/ml)	MIC_MXF+R_ (μg/ml)	No. isolates	GyrA			GyrB	ParC					ParE
			S81	E85	*S114*	P454	*S52*	*N91*	D78	S79	D83	D435
< 1	0.064–0.19	289	-	-	-	-	-	-	-	-	-	-
(*n* = 311)		10	-	-	*G*	-	-	-	-	-	-	-
		6	-	-	*G*	-	*G*	*D*	-	-	-	-
		2	-	-	-	-	-	*D*	-	-	-	-
		2	-	-	-	-	*G*	*D*	-	-	-	-
		1	-	-	*G*	-	*G*	-	-	-	-	-
		1	F	-	-	-	-	-	-	-	-	-
≤ 2	0.125–0.25*	47	-	-	-	-	-	-	-	-	-	-
(*n* = 80)		12	-	-	-	-	-	-	-	F	-	-
		1	-	-	-	-	-	-	N	-	-	-
		1	F	-	-	-	-	-	-	-	-	-
		1	-	-	-	-	-	*D*	-	F	-	-
		2	-	-	-	-	-	-	-	-	N/Y	-
		1	-	-	-	-	-	-	-	F	-	K
		1	-	-	*G*	-	*G*	*D*	-	F	-	-
		5	-	-	*G*	-	-	-	-	-	-	-
		5	-	-	*G*	-	*G*	*D*	-	-	-	-
		4	-	-	*G*	-	-	*D*	-	-	-	-
2–4	0.19–1	3	-	-	-	-	-	-	-	-	-	-
(*n* = 12)		4	-	-	-	-	-	-	-	Y/F	-	-
		1	F	-	-	-	-	-	-	-	-	N
		1	-	-	-	-	-	*D*	-	F	-	-
		1	-	-	-	-	-	-	-	-	G	-
		1	-	-	*G*	-	-	-	-	-	-	-
		1	-	-	*G*	-	*G*	*D*	-	-	-	-
		1	-	-	-	-	-	-	-	-	-	-
		3	F	-	-	-	-	-	-	-	-	-
≥4	1–32	1	F	-	-	-	-	-	-	-	-	N
(*n* = 29)		12	F	-	-	-	-	-	-	F/Y	-	-
		2	F	-	-	-	-	-	-	-	G/Y	-
		2	-	-	*G*	-	-	*D*	-	F	-	-
		1	Y	-	*G*	-	-	*D*	-	Y	-	-
		1	G	-	-	-	-	-	-	-	-	-
		2	-	-	-	-	-	-	-	-	N	-
		2	-	-	-	-	-	-	-	F/Y	-	-
		1	-	K	-	-	-	-	-	Y	-	-
		1	L	-	*G*	S	-	-	-	-	-	-

Only shown are amino acid substitutions involved in resistance and recombination with the viridans group. -, wild type. Identified mutations not involved in resistance were **GyrA**: M99I (*n* = 3), G112D (*n* = 2), G103S, L152R, L155F, L155V, L154F, L157F(*n* = 1); **GyrB:** G434R (*n* = 4), D435N, S466G, F480L (*n* = 1); **ParC**: K137N (*n* = 64), K57T (*n* = 11), R95C/G (*n* = 3), E134D, E135D (*n* = 1); **ParE**: I460V (*n* = 232), H534L/Q (*n* = 6), I493L (*n* = 3), A532T/V (*n* = 2), D435N, l431S, I493L, A468T, Y481H, K448P, Q420P (*n* = 1).

*0.38 for the isolate with S81F

Classical QRDR mutations were retrieved in GyrA at positions 81 (*n* = 24) and 85 (*n* = 1), ParC positions 78 (*n* = 1), 79 (*n* = 38) and 83 (*n* = 7), and ParE position 435 (*n* = 3). These topoisomerase mutations were found in varying combinations, arguing against clonal expansion of FQ-R (Table 2). To investigate this hypothesis, we performed CST typing on 54 FQ-R isolates which showed a wide variety of associated serotypes (S3 Table). In concordance to previous studies, isolates carrying mutations in both topoisomerases unequivocally displayed high-level resistance to CIP (MIC_CIP+R_ > 12 μg/mL). In contrast, strains with sole mutations in ParC (50.9% of isolates with mutated QRDR) or GyrA (13.7%) display more variable MIC_CIP+R_ values. For example, four strains carrying a GyrA S81F/G mutation displayed a MIC_CIP+R_ of 32 μg/mL (e.g., 13C28), whereas other strains with the same mutation (*e*.*g*., 05A05 and 04L17) only reached 0.5–1 μg/mL.

Finally, some interesting genotypes were observed. For example, we identified a very rare GyrB mutation (P454S) in isolate 05A20 which, in combination with GyrA S81L and wild-type Topoisomerase IV, was correlated with full resistance to MXF (MIC_MXF+R_ = 32 μg/mL) (Table 2). An even more noticeable result was that some isolates with complete wild-type QRDRs (e.g., 13C24, 11I08) were nevertheless fully resistant to CIP, even in the presence of reserpine (Table 2).

### Efflux pump expression analysis

Next, we assessed the contribution of PmrA and PatAB efflux transporters to FQ-R by measuring early-log expression levels of *patA*, *patB* and *pmrA* in 94 *S*. *pneumoniae* isolates grown in the absence of antibiotics, in comparison to the control strain *S*. *pneumoniae* ATCC 49619 [27,28]. Strains were selected based on various susceptibilities to reserpine, i.e. displaying either no (n = 2), 1- (n = 8), 2- (n = 20), 3- (n = 24), 4-(n = 25) or ≥5 (n = 14) fold MIC_CIP_ reductions in the presence of this efflux pump inhibitor. The results are shown in Table 3.

**Table 3:** Expression analyses of a selection of *S*. *pneumoniae* strains, with inclusion of the QRDR sequences and phenotypic FQ-R analyses.

Strain Id.	Topoisomerase mutations				MIC_CIP + reserpine_ (μg/ml)^a^		MIC_MXF + reserpine_ (μg/ml)^a^		Gene expression		
	GyrA	GyrB	ParC	ParE	0	20	0	20	*pmrA*	*patA*	*patB*
**07A40**	S81F	wt	S79F	wt	>32	>32	3	2	0.6 ± 0.2	11.2 ± 4.3	0.7 ± 0.2
**10N11**	S81F	wt	S79Y	wt	>32	>32	8	4	0.3 ± 0.1	10.4 ± 2.9	281.5 ± 37.7
**12K23**	wt	wt	wt	I460V	1	0.75	0.125	0.094	1.7 ± 1.1	2.8 ± 1.8	19.9 ± 12.4
**ATCC**	wt	wt	wt	wt	0.5	0.35	0.064	0.064	1	1	1
**99H18**	wt	wt	wt	I460V	1.5	1	0.19	0.125	13.1 ± 8.5	0.2 ± 0.1	10.2 ± 7.7
**12M03**	wt	wt	wt	I460V	1.5	1	0.125	0.125	1.3 ± 0.4	1.3 ± 0.6	10.0 ± 3.5
**08 E03**	wt	wt	R95C	wt	0.75	0.5	0.094	0.064	1.0 ± 0.2	1.9 ± 0.6	0.7 ± 0.3
**05A07**	wt	S466G	K57T	I460V	0.75	0.38	0.125	0.094	0.2 ± 0.1	1.0 ± 0.2	10.3 ± 2.8
**11I08**	wt	wt	wt	I460V	32	16	1	1	1.0 ± 0.3	0.5 ± 0.3	0.7 ± 0.1
**11A23**	wt	wt	D83N	I460V	>32	16	1.5	1.5	0.3 ± 0.1	3.3 ± 0.8	39.2 ± 10.7
**08A02**	wt	wt	wt	wt	0.5	0.25	0.125	0.064	0.4 ± 0.06	1.1 ± 0.1	0.4 ± 0.1
**09K10**	wt	wt	wt	I460V	1	0.5	0.094	0.094	3.8 ± 1.7	0.3 ± 0.1	0.3 ± 0.1
**13L15**	wt	wt	wt	I460V	1.5	0.75	0.125	0.125	1.7 ± 0.8	1.8 ± 0.9	13.7 ± 7.0
**11I10**	wt	wt	wt	I460V	1	0.5	0.125	0.125	0.8 ± 0.2	1.9 ± 0.6	16.9 ± 5.5
**09K03**	wt	wt	wt	I460V	1	0.5	0.094	0.094	1.6 ± 0.4	0.2 ± 0.04	0.7 ± 0.2
**97G03**	wt	wt	S79F	A532T	2	1	0.19	0.125	4.1 ± 1.3	0.1 ± 0.05	9.1 ± 3.3
**11I29**	wt	wt	wt	I460V	1.5	0.75	0.125	0.125	0.9 ± 0.4	1.5 ± 0.8	24.4 ± 10.3
**10A15**	wt	wt	wt	wt	1	0.5	0.125	0.125	1.1 ± 0.4	2.3 ± 0.7	68.0 ± 22.0
**07M27**	wt	wt	R95C	wt	1	0.5	0.125	0.125	1.6 ± 0.9	4.1 ± 2.5	0.2 ± 0.1
**09L20**	wt	wt	wt	I460V	1	0.5	0.125	0.094	6.7 ± 5.4	3.6 ± 3.1	2.1 ± 1.2
**09N30**	wt	wt	wt	I460V	1.5	0.75	0.064	0.064	2.6 ± 0.9	9.1 ± 3.4	8.2 ± 2.8
**04F27**	wt	wt	wt	I460V	1	0.38	0.094	0.064	1.9 ± 0.9	1.8 ± 0.3	11.8 ± 2.3
**05C40**	wt	wt	K137N	I460V	1	0.38	0.125	0.094	5.3 ± 3.5	4.5 ± 2.3	25.4 ± 13.0
**01H28**	S81F	wt	K137N	I460V	32	12	3	2	10.7 ± 4.1	1.6 ± 0.4	15.1 ± 3.3
**13A22**	wt	wt	wt	I460V	4	1.5	0.125	0.125	0.5 ± 0.3	2.6 ± 1.5	14.8 ± 8.5
**09K16**	wt	wt	wt	I460V	2	0.75	0.125	0.125	1.8 ± 1.1	0.3 ± 0.1	0.4 ± 0.5
**11O31**	S114G	wt	wt	wt	4	1.5	0.125	0.125	5.7 ± 5.7	14.3 ± 12.6	24.3 ± 3.9
**07O07**	S114G	wt	S79F/N91D	I460V	16	6	0.25	0.25	0.6 ± 0.03	1.8 ± 0.0	0.5 ± 0.1
**97B14**	L154F	wt	wt	I460V	1.5	0.5	0.094	0.094	2.4 ± 0.8	0.08 ± 0.03	9.8 ± 4.1
**04A10**	wt	wt	wt	I460V	1.5	0.5	0.125	0.125	9.3 ± 3.3	0.5 ± 0.2	0.3 ± 0.2
**97I27**	wt	wt	wt	I460V	1.5	0.5	0.125	0.094	8.4 ± 3.7	0.1 ± 0.04	9.7 ± 4.2
**04A25**	wt	wt	wt	I460V	3	1	0.19	0.19	9.4 ± 5.4	0.4 ± 0.2	0.4 ± 0.2
**13L23**	wt	wt	wt	I460V	3	1	0.19	0.19	1.7 ± 0.5	14.8 ± 4.4	48.4 ± 10.5
**06N06**	wt	wt	wt	I460V	1.5	0.5	0.125	0.125	1.1 ± 0.2	2.4 ± 0.6	0.2 ± 0.1
**13B09**	wt	wt	wt	I460V	1.5	0.5	0.094	0.094	0.8 ± 0.5	1.0 ± 0.5	9.6 ± 6.1
**95B18**	wt	wt	wt	wt	1.5	0.5	0.125	0.125	1.4 ± 0.5	42.4 ± 15.0	198.6 ± 71.1
**4E+13**	wt	wt	wt	I460V	1.5	0.5	0.125	0.125	3.7 ± 1.5	0.5 ± 0.3	0.1 ± 0.1
**95C17**	wt	wt	wt	I460V	0.75	0.25	0.094	0.094	2.0 ± 0.2	0.3 ± 0.1	2.8 ± 1.1
**13E324**	S114G	wt	wt	I460V	1.5	0.5	0.125	0.125	0.8 ± 0.4	1.9 ± 0.9	9.2 ± 4.6
**13L14**	wt	wt	wt	I460V	3	1	0.19	0.19	1.2 ± 0.5	2.5 ± 1.1	13.3 ± 5.7
**04L07**	wt	wt	wt	I460V	1.5	0.5	0.094	0.094	3.9 ± 1.9	1.3 ± 0.6	0.5 ± 0.2
**06A24**	wt	wt	wt	I460V	1.5	0.5	0.125	0.125	0.6 ± 0.2	2.4 ± 1.0	0.5 ± 0.2
**10I25**	wt	wt	wt	I460V	3	1	0.125	0.125	1.2 ± 0.6	6.6 ± 2.2	72.2 ± 45.7
**04C04**	wt	wt	wt	I460V	1.5	0.5	0.094	0.094	4.3 ± 1.8	2.8 ± 1.4	1.3 ± 0.5
**95C08**	wt	wt	S52G/N91D	I460V	1.5	0.5	0.19	0.19	1.6 ± 1.4	6.2 ± 4.6	32.9 ± 24.2
**04L24**	wt	wt	K137N	I460V	1.5	0.5	0.094	0.094	4.5 ± 0.8	1.8 ± 0.3	0.5 ± 0.1
**10L18**	wt	wt	wt	I460V	1.5	0.5	0.25	0.25	1.1 ± 0.3	1.5 ± 0.5	16.1 ± 5.3
**13A12**	wt	wt	wt	I460V	1.5	0.5	0.125	0.125	1.4 ± 0.4	3.5 ± 0.3	25.0 ± 3.0
**09O15**	wt	wt	wt	I460V	0.75	0.25	0.064	0.064	1.2 ± 0.1	0.5 ± 0.07	0.4 ± 0.07
**06O04**	wt	wt	wt	wt	1.5	0.38	0.094	0.094	0.7 ± 0.4	2.0 ± 1.1	0.3 ± 0.1
**08J09**	wt	wt	wt	I460V	2	0.5	0.125	0.094	0.7 ± 0.08	0.15 ± 0.0	0.2 ± 0.1
**13B01**	wt	wt	wt	I460V	3	0.75	0.19	0.19	0.9 ± 0.2	1.5 ± 0.3	8.6 ± 2.6
**06A08**	S114G	wt	wt	A496T	2	0.5	0.094	0.125	3.6 ± 2.4	2.3 ± 1.4	12.2 ± 7.9
**99H17**	wt	wt	wt	wt	0.5	0.125	0.064	0.032	7.7 ± 3.0	0.09 ± 0.04	8.9 ± 3.7
**06J35**	wt	wt	wt	wt	2	0.5	0.125	0.125	1.4 ± 0.8	1.4 ± 0.7	11.0 ± 5.8
**12M02**	wt	wt	S79F/ N91D	I460V	4	1	0.125	0.125	4.1± 0.6	0.03 ± 0.01	10.8 ± 1.8
**95B16**	S114G	wt	S52G/N91D	wt	4	1	0.25	0.19	2.7 ± 0.8	0.04 ± 0.0	4.8 ± 1.1
**99A09**	wt	wt	wt	I460V	2	0.5	0.19	0.125	11.0 ± 9.6	0.2 ± 0.1	9.3 ± 8.4
**08G28**	wt	wt	wt	I460V	3	0.75	0.125	0.094	0.3 ± 0.03	0.4 ± 0.03	0.2 ± 0.0
**06J37**	wt	wt	wt	I460V	2	0.5	0.125	0.125	1.4 ± 0.4	1.6 ± 0.9	5.8 ± 0.7
**01H21**	S81F	wt	K137N	D435N	16	4	1.5	1	9.9 ± 3.3	3.0 ± 0.6	13.3 ± 4.5
**03L14**	wt	wt	wt	I460V	2	0.5	0.094	0.094	4.4 ± 2.4	0.7 ± 0.4	0.3 ± 0.2
**05A28**	S114G	wt	S52G/N91D	wt	4	1	0.19	0.19	0.02 ± 0.0	0.8 ± 0.3	0.7 ± 0.2
**08G25**	wt	G434R	wt	I460V	2	0.5	0.125	0.094	0.2 ± 0.02	2.4 ± 0.4	0.6 ± 0.1
**95F08**	wt	wt	wt	I460V	2	0.5	0.094	0.094	4.5 ± 2.7	2.0 ± 1.1	53.7 ± 28.7
**07H01**	wt	wt	S79F	I460V	4	1	0.125	0.125	1.5 ± 0.9	21.4 ± 11.9	5.6 ± 3.0
**08G26**	wt	wt	wt	I460V	2	0.5	0.094	0.094	1.1 ± 0.5	3.0 ± 1.5	0.4 ± 0.2
**08O21**	wt	wt	K137N	I460V	2	0.5	0.125	0.125	1.9 ± 0.6	6.5 ± 1.3	1.6 ± 0.3
**05D34**	wt	wt	wt	I460V	4	1	0.125	0.125	7.0 ± 0.4	0.3 ± 0.04	2.8 ± 0.1
**10K19**	wt	wt	wt	I460V	6	1.5	0.125	0.125	0.5 ± 0.05	11.1 ± 1.1	208.7 ± 34.9
**97A11**	wt	wt	wt	I460V	2	0.5	0.125	0.125	1.8 ± 0.6	1.0 ± 0.3	121.4 ± 49.5
**97B21**	wt	wt	wt	wt	2	0.38	0.125	0.094	1.7 ± 0.8	0.2 ± 0.1	18.7 ± 10.3
**05A34**	S114G	wt	S52G/N91D	wt	4	0.75	0.19	0.19	0.3 ± 0.1	0.3 ± 0.1	0.04 ± 0.01
**99D02**	wt	wt	K137N	I460V	4	0.75	0.094	0.064	1.1 ± 0.4	0.4 ± 0.2	34.9 ± 12.1
**99J08**	wt	wt	wt	I460V	4	0.75	0.125	0.125	3.2 ± 2.2	1.4 ± 1.1	134.0 ± 96.9
**97B25**	wt	wt	ND	wt	24	4	0.38	0.38	2.2 ± 1.3	0.1 ± 0.08	10.0 ± 5.9
**08 E15**	wt	wt	wt	I460V	6	1	0.125	0.094	3.5 ± 2.1	0.2 ± 0.01	1.0 ± 0.6
**11A17**	wt	wt	wt	I460V	3	0.5	0.125	0.125	0.4 ± 0.1	9.9 ± 2.4	79.7 ± 21.4
**08L06**	wt	wt	wt	I460V	3	0.5	0.094	0.094	1.1 ± 0.2	0.3 ± 0.07	0.3 ± 0.1
**01C06**	wt	wt	K137N	I460V	3	0.38	0.125	0.125	11.4 ± 4.7	1.3 ± 0.4	12.2 ± 4.4
**99H10**	wt	wt	wt	wt	1.5	0.19	0.094	0.094	8.2 ± 2.1	0.5 ± 0.08	18.9± 3.1
**04A24**	wt	wt	wt	I460V	8	1	0.19	0.125	5.6 ± 3.4	0.4 ± 0.1	15.5 ± 4.9
**03N11**	wt	wt	S79Y	I460V	16	2	0.19	0.19	8.0 ± 3.3	1.4 ± 0.2	41.1 ± 8.7
**10A19**	wt	wt	wt	I493L	4	0.5	0.125	0.094	0.6 ± 0.3	0.0 ± 0.00	73.2 ± 50.1
**13F15**	S114G	wt	wt	wt	8	1	0.19	0.19	1.3 ± 0.3	1.1 ± 0.2	10.8 ± 2.6
**99G11**	wt	wt	wt	I460V	6	0.75	0.125	0.125	9.7 ± 2.4	2.9 ± 0.6	19.5 ± 0.7
**11A27**	S114G	wt	N91D	I493L	8	1	0.19	0.125	2.8 ± 1.7	3.1 ± 1.9	32.0 ± 17.3
**09K15**	wt	wt	wt	I460V	4	0.5	0.094	0.094	1.2 ± 0.3	0.6 ± 0.2	0.7 ± 0.2
**01C35**	S114G	wt	S79Y/N91D	I460V	16	2	0.125	0.125	16.3 ± 5.9	3.4 ± 1.1	0.15 ± 0.05
**01G18**	wt	wt	wt	I460V	4	0.38	0.125	0.094	14.3 ± 3.8	3.5 ± 0.3	30.8 ± 4.9
**03 K18**	wt	wt	wt	I460V	8	0.75	0.125	0.125	6.2 ± 5.4	13.5 ± 16.6	9.8 ± 11.4
**03 L23**	wt	wt	S79F	D435K	32	2	0.19	0.19	7.9 ± 4.1	11.5 ± 5.8	153.9 ± 73.3
**06H02**	S114G	wt	wt	wt	>32	2	0.19	0.125	0.8 ± 0.3	2.0 ± 1.5	0.3 ± 0.1
**13G08**	S114G	wt	N91D	wt	16	1	0.19	0.19	1.5 ± 0.6	5.4 ± 2.4	34.0 ± 15.8
**03L28**	wt	wt	K137N	I460V	>32	1.5	0.125	0.125	11.6 ± 3.3	1.7 ± 0.4	18.6 ± 4.6
**99J16**	wt	wt	wt	I460V	8	0.38	0.094	0.094	6.5 ± 0.4	6.0 ± 1.3	56.5 ± 13.6

^a^ Based on E-test.

Since putative highly different genetic backgrounds preclude reliable strain-to-strain comparison, we performed Kruskal-Wallis testing (with Dunn’s multiple comparison test) on three sample groups of strains either susceptible, intermediate or resistant to CIP (S1 Fig). This non-parametric method showed no statistically significant differences in *pmrA* expression among the three groups. In contrast, both *patA* and *patB* expression was significantly upregulated in CIP-resistant, but not in CIP-intermediate *S*. *pneumoniae* strains. Although this correlated with the observed susceptibility to reserpine, the levels of reserpine-mediated MIC reductions varied strongly among strains with similar transcript levels. For example, strains 99J16 and 13L23 both strongly overexpressed *patA* (6.0±1.3 and 14.8±4.4, resp.) and *patB* (56.5±13.6 and 48.4±10.5, resp.), but showed a sixteen- vs. threefold reduction in MIC_CIP_ in the presence of the efflux pump inhibitor.

Conversely, strain 10N11 also overexpressed *patA* and *patB* but showed no reserpine-dependent reduction of MIC_CIP_ (Table 3). Notably, in 12 and 19 isolates only *patA* or *patB* were overexpressed, respectively, arguing against uniform operon coregulation for these genes. Finally, in 17.0% of the reserpine-susceptible strains both *patA* and *patB* were downregulated. Since the heterogeneous PatAB pump requires both functional subunits to be functional [36], these two last observations strongly indicate the presence of other reserpine-sensitive systems involved in FQ-R.

Constitutive induction of *patA* has very recently been correlated to disruption of the transcriptional terminator of the upstream *hexA* gene [22,24]. We therefore sequenced this upstream region in 103 isolates. Although none of previous described mutations were retrieved, we identified six novel mutations: C(-41)T, G(-40)A, G(-46)T, G(-48)A, G(-49)A and C(-28)T. Each of these mutations could be related to decreased hairpin stability (ΔG increases > 3.2 kCal/mol), leading to increased transcription. The A(-52)G mutation was found not to play a role in *patA* regulation. It is important to note that these mutations were found in only a fraction (15.5%) of the isolates which overexpress *patA*, implying that terminator disruption is only a minor regulatory mechanism in the isolates under study.

## Discussion

At the introduction of the respiratory fluoroquinolones LVX and MXF in the treatment of (a)typical pneumonia, there was concern that while treatment success in the short term could be enhanced, highly FQ-R *S*. *pneumoniae* strains would emerge by accumulation of additional QRDR mutations [37]. The continued high use of CIP for specific respiratory indications, such as the treatment of bronchial infections in cystic fibrosis patients [13], poses an additional risk factor to select for first-step ParC mutations which precede the ones in GyrA under CIP selective pressure. Moreover, the continuous exposure to sub-MIC levels of CIP and levofloxacin has been shown to select for efflux overexpression [38].

In our surveillance data on FQ-R among Belgian non-invasive *S*. *pneumoniae* isolates (1995–2014), some evidence points in this direction. From 2011 onwards, we observe a trend towards increased resistance to CIP and ofloxacin, and (although only visible at the MIC_50_ level) also for LVX. Our data from CST typing clearly indicates no clonal spread of CIP-R isolates, and thereby suggests there is no direct influence of vaccination campaigns on FQ-R in non-invasive pneumococci. The preference of first-step mutations in ParC is reflected by the 4:1 ratio of single QRDR mutations in the Topoisomerase IV subunits compared to the DNA Gyrase. Although a similar increase in CIP resistance was reported in Canada [10], this was not confirmed in other surveillance studies covering Europe, North America or Asia [8,9]. In contrast, resistance to MXF was only sporadic and globally minimal. As these trends are still very recent, it is critical that resistance rates and their changes are continuously monitored in the near future.

The exceptional case of high-level resistance to MXF (MIC_MXF+R_ of 32 μg/ml) was associated with a GyrB P454S mutation, combined with a mutated GyrA (S81L) subunit but a wild-type ParC/E. Notably, a Chinese group recently reported both ParE_P454S as GyrB_P454S to be associated with MXF resistance in combination with dually mutated GyrA and ParC residues [39,40]. However, our observation of a wild-type Topoisomerase IV QRDR region is important, as it contrasts with the current model which states that mutations in both topoisomerases are a prerequisite for high-level MXF resistance.

Analysis of the contribution of efflux pumps to pneumococcal FQ-R revealed no significant upregulation of the Major Facilitator PmrA in CIP resistant strains. We did observe varying constitutive expression of *pmrA* among clinical isolates, which has been shown before [16], but its contribution to drug resistance in non-invasive pneumococcal strains remains unclear. In contrast, non-parametric analyses showed marked higher expression of the ABC efflux pump PatAB associated with decreasing CIP susceptibility. Unfortunately, the underlying regulatory mechanism behind this upregulation remains unexplored. Although we found novel disruptive mutations in upstream transcriptional terminator sequences [22–24], this mechanism seems rather rare among clinical isolates as the large majority has wild-type upstream regions. A *patA/B* repressor has not been found or seems deleted in comparison to similar operons in related bacteria [41]. Various other levels of regulation can be envisioned at the post-transcriptional, translational or post-translational level.

Another important finding of this study is related to strains which seem deprived of known molecular FQ-R mechanisms, but yet display a resistant phenotype. For example, isolate 11I08 displays a wild-type QRDR yet a MIC_CIP+R_ of 24 μg/mL. A possibility is the involvement of chromosomally encoded qnr-like proteins, which shield topoisomerases from invading fluoroquinolones [42]. In contrast, many reserpine-susceptible strains did not express PatAB pumps, implying the involvement of other reserpine-sensitive efflux mechanisms in pneumococcus. This can be either novel efflux pumps, like the recently identified DinF transporter [43], or any of the five transporter genes found to be consistently induced by fluoroquinolones [44]. In any case, isolates with elevated MICs but without defined resistance mechanism are also commonly reported in other studies [3, 17, 25], and deserve more attention in the future.

We acknowledge limitations of the presented *patAB* expression studies. First of all, putative gene duplication of *patA* [23] could not be detected with the applied methods. Moreover, although we assessed constitutive gene expression, it has been shown that expression is quickly upregulated upon exposure to CIP, with *patA* being more strongly upregulated than *patB* [19,40]. This might level out the difference we observed in basal expression levels between both genes.

In conclusion, 15 years after the introduction of respiratory fluoroquinolones, we observe a concerning rise in resistance among non-invasive pneumococci. MXF remains a very potent drug with minimal level of resistance, but a combination of rare mutations in the DNA Gyrase was associated with full resistance to this compound. While target topoisomerase mutations and efflux pump (over)expression clearly contribute to FQ-R, we add novel isolates to the existing collection of strains deprived of known molecular mechanisms of fluoroquinolone resistance. It would be of great value to bring these clinical isolates together, and unravel their resistance mechanisms through a profound, comparative molecular characterization at the genomic, transcriptomic and proteomic level.

## Supporting Information

S1 Fig Gene expression analyses. (DOCX)

S1 Table MIC distribution of the reference strains used in the broth microdilution experiments. (DOCX)

S2 Table Oligonucleotides used in this study. (DOCX)

S3 Table Results of CST typing. (DOCX)

S4 Table QRDR sequencing and MIC determination of a selection of 422 pneumococcal strains. (DOCX)

## References

[1] Bridy-Pappas AE, Margolis MB, Center KJ, Isaacman DJ. Streptococcus pneumoniae: description of the pathogen, disease epidemiology, treatment, and prevention. Pharmacotherapy 2005; 25: 1193–1212. 16164394 10.1592/phco.2005.25.9.1193

[2] Liñares J, Ardanuy C, Pallares R, Fenoll A. Changes in antimicrobial resistance, serotypes and genotypes in Streptococcus pneumoniae over a 30-year period. Clin Microbiol Infect. 2010; 16: 402–410. 10.1111/j.1469-0691.2010.03182.x 20132251

[3] Adam HJ, Hoban DJ, Gin AS, Zhanel GG. Association between fluoroquinolone usage and a dramatic rise in ciprofloxacin-resistant Streptococcus pneumoniae in Canada, 1997–2006. Int J Antimicrob Agents 2009; 3: 82–85. 10.1016/j.ijantimicag.2009.02.002 19342204

[4] Zhanel GG, Ennis K, Vercaigne L, Walkty A, Gin AS, Embil J, et al A critical review of the fluoroquinolones: focus on respiratory infections. Drugs 2002; 62: 13–59. 11790155 10.2165/00003495-200262010-00002

[5] Hooper DC. Mode of action of fluoroquinolones. Drugs 1999; 58 Suppl 2: 6–10. 10553698 10.2165/00003495-199958002-00002

[6] Janoir C, Zeller V, Kitzis MD, Moreau NJ, Gutmann L. High-level fluoroquinolone resistance in Streptococcus pneumoniae requires mutations in parC and gyrA. Antimicrob Agents Chemother. 1996; 40: 2760–4. 9124836 10.1128/aac.40.12.2760 PMC163617

[7] Jorgensen JH, Weigel LM, Ferraro MJ, Swenson JM, Tenover FC. Activities of newer fluoroquinolones against Streptococcus pneumoniae clinical isolates including those with mutations in the gyrA, parC, and parE loci. Antimicrob Agents Chemother. 1999; 43: 329–34. 9925527 10.1128/aac.43.2.329 PMC89072

[8] Jones ME, Sahm DF, Martin N, Scheuring S, Heisig P, Thornsberry C, et al Prevalence of gyrA, gyrB, parC, and parE mutations in clinical isolates of Streptococcus pneumoniae with decreased susceptibilities to different fluoroquinolones and originating from Worldwide Surveillance Studies during the 1997–1998 respiratory season. Antimicrob Agents Chemother. 2000; 44: 462–466. 10639387 10.1128/aac.44.2.462-466.2000 PMC89708

[9] Patel SN, Melano R, McGeer A, Green K, Low DE. Characterization of the quinolone resistant determining regions in clinical isolates of pneumococci collected in Canada. Ann Clin Microbiol Antimicrob. 2010; 9:3 10.1186/1476-0711-9-3 20082699 PMC2823643

[10] Li X, Zhao X, Drlica K. Selection of Streptococcus pneumoniae mutants having reduced susceptibility to moxifloxacin and levofloxacin. Antimicrob Agents Chemother. 2002; 46: 522–524. 11796368 10.1128/AAC.46.2.522-524.2002 PMC127057

[11] Smith HJ, Walters M, Hisanaga T, Zhanel GG, Hoban DJ. Mutant prevention concentrations for single-step fluoroquinolone-resistant mutants of wild-type, efflux-positive, or ParC or GyrA mutation-containing Streptococcus pneumoniae isolates. Antimicrob Agents Chemother. 2004; 48: 3954–3958. 15388458 10.1128/AAC.48.10.3954-3958.2004 PMC521923

[12] Maeda Y, Murayama M, Goldsmith CE, Coulter WA, Mason C, Millar BC, et al Molecular characterization and phylogenetic analysis of quinolone resistance-determining regions (QRDRs) of gyrA, gyrB, parC and parE gene loci in viridans group streptococci isolated from adult patients with cystic fibrosis. J Antimicrob Chemother. 2011; 66: 476–486. 10.1093/jac/dkq485 21193474

[13] Jumbe NL, Louie A, Miller MH, Liu W, Deziel MR, Tam VH, et al Quinolone efflux pumps play a central role in emergence of fluoroquinolone resistance in Streptococcus pneumoniae. Antimicrob Agents Chemother. 2006; 50: 310–317. 16377702 10.1128/AAC.50.1.310-317.2006 PMC1346791

[14] Gill MJ, Brenwald NP, Wise R. Identification of an efflux pump gene, pmrA, associated with fluoroquinolone resistance in Streptococcus pneumoniae. Antimicrob Agents Chemother. 1999; 43: 187–189. 9869592 10.1128/aac.43.1.187 PMC89047

[15] Piddock LJ, Johnson MM, Simjee S, Pumbwe L. Expression of efflux pump gene pmrA in fluoroquinolone-resistant and -susceptible clinical isolates of Streptococcus pneumoniae. Antimicrob Agents Chemother. 2002; 46: 808–812. 11850265 10.1128/AAC.46.3.808-812.2002 PMC127475

[16] Garvey MI, Baylay AJ, Wong RL, Piddock LJ. Overexpression of patA and patB, which encode ABC transporters, is associated with fluoroquinolone resistance in clinical isolates of Streptococcus pneumoniae. Antimicrob Agents Chemother. 2011; 55: 190–196. 10.1128/AAC.00672-10 20937787 PMC3019642

[17] Garvey MI, Piddock LJ. The efflux pump inhibitor reserpine selects multidrug-resistant Streptococcus pneumoniae strains that overexpress the ABC transporters PatA and PatB. Antimicrob Agents Chemother. 2008; 52: 1677–1685. 10.1128/AAC.01644-07 18362193 PMC2346654

[18] El Garch F, Lismond A, Piddock LJ, Courvalin P, Tulkens PM, Van Bambeke F. Fluoroquinolones induce the expression of patA and patB, which encode ABC efflux pumps in Streptococcus pneumoniae. J Antimicrob Chemother. 2010; 65: 2076–2082. 10.1093/jac/dkq287 20709735

[19] Avrain L, Garvey M, Mesaros N, Glupczynski Y, Mingeot-Leclercq MP, Piddock LJ, et al Selection of quinolone resistance in Streptococcus pneumoniae exposed in vitro to subinhibitory drug concentrations. J Antimicrob Chemother. 2007; 60: 965–972. 17693451 10.1093/jac/dkm292

[20] Robertson GT, Doyle TB, Lynch AS. Use of an efflux-deficient streptococcus pneumoniae strain panel to identify ABC-class multidrug transporters involved in intrinsic resistance to antimicrobial agents. Antimicrob Agents Chemother. 2005; 49: 4781–4783. 16251330 10.1128/AAC.49.11.4781-4783.2005 PMC1280156

[21] Baylay AJ, Ivens A, Piddock LJ. A novel gene amplification causes upregulation of the PatAB ABC transporter and fluoroquinolone resistance in Streptococcus pneumoniae. Antimicrob Agents Chemother. 2015; 59: 3098–108. 10.1128/AAC.04858-14 25779578 PMC4432121

[22] Baylay AJ, Piddock LJ. Clinically relevant fluoroquinolone resistance due to constitutive overexpression of the PatAB ABC transporter in Streptococcus pneumoniae is conferred by disruption of a transcriptional attenuator. J Antimicrob Chemother. 2015; 70: 670–679. 10.1093/jac/dku449 25411187 PMC4319486

[23] Lupien A, Gingras H, Bergeron MG, Leprohon P, Ouellette M. Multiple mutations and increased RNA expression in tetracycline-resistant Streptococcus pneumoniae as determined by genome-wide DNA and mRNA sequencing. J Antimicrob Chemother. 2015; 70: 1946–1959. 10.1093/jac/dkv060 25862682 PMC4472328

[24] Lupien A, Billal DS, Fani F, Soualhine H, Zhanel GG, Leprohon P, et al Genomic characterization of ciprofloxacin resistance in a laboratory-derived mutant and a clinical isolate of Streptococcus pneumoniae. Antimicrob Agents Chemother. 2013; 57: 4911–4919. 10.1128/AAC.00418-13 23877698 PMC3811476

[25] Said MA, Johnson HL, Nonyane BAS, Deloria-Knoll M, O'Brien KL, AGEDD Adult Pneumococcal Burden Study Team, et al Estimating the burden of pneumococcal pneumonia among adults: a systematic review and meta-analysis of diagnostic techniques. PLoS ONE 2013; 8: e60273 10.1371/journal.pone.0060273 23565216 PMC3615022

[26] Ubukata K, Asahi Y, Yamane A, Konno M. Combinational detection of autolysin and penicillin-binding protein 2B genes of Streptococcus pneumoniae by PCR. J Clin Microbiol. 1996; 34: 592–6. 8904421 10.1128/jcm.34.3.592-596.1996 PMC228853

[27] Elberse KE, van de Pol I, Witteveen S, van der Heide HG, Schot CS, van Dijk A, et al Population structure of invasive Streptococcus pneumoniae in The Netherlands in the pre-vaccination era assessed by MLVA and capsular sequence typing. PLoS One. 2011; 6: e20390 10.1371/journal.pone.0020390 21637810 PMC3102707

[28] Blondeau JM, Vaughan D, Laskowski R, Borsos S; Canadian Antimicrobial Study Group. Susceptibility of Canadian isolates of Haemophilus influenzae, Moraxella catarrhalis and Streptococcus pneumoniae to oral antimicrobial agents. Int J Antimicrob Agents. 2001; 17:457–64. 11397615 10.1016/s0924-8579(01)00334-x

[29] Firsov AA, Alferova IV, Smirnova MV, Lubenko IY, Portnoy YA, Zinner SH. Comparative pharmacodynamics of the new fluoroquinolone ABT492 and levofloxacin with Streptococcus pneumoniae in an in vitro dynamic model. Int J Antimicrob Agents. 2005; 25: 409–13. 15848296 10.1016/j.ijantimicag.2005.02.004

[30] Pfaffl MW. Quantification strategies in real-time PCR In: Bustin SA, editor. A-Z of quantitative PCR, La Jolla; 2004; p. 87–112.

[31] Dang TN, Srinivasan U, Britt Z, Marrs CF, Zhang L, Ki M, et al Efflux-mediated resistance identified among norfloxacin resistant clinical strains of group B Streptococcus from South Korea. Epidemiol Health. 2014; 36: e2014022 10.4178/epih/e2014022 25322878 PMC4258715

[32] Kaatz GW, Seo SM, O'Brien L, Wahiduzzaman M, Foster TJ. Evidence for the existence of a multidrug efflux transporter distinct from NorA in Staphylococcus aureus. Antimicrob Agents Chemother. 2000; 44: 1404–6. 10770791 10.1128/aac.44.5.1404-1406.2000 PMC89884

[33] Johnson AP, Warner M, Livermore DM. Activity of linezolid against multi-resistant gram-positive bacteria from diverse hospitals in the United Kingdom. J Antimicrob Chemother. 2000; 45:225–30. 10660506 10.1093/jac/45.2.225

[34] Tubau F, Fernández-Roblas R, Liñares J, Martín R, Soriano F. In vitro activity of linezolid and 11 other antimicrobials against 566 clinical isolates and comparison between NCCLS microdilution and Etest methods. J Antimicrob Chemother. 2001; 47:675–80. 11328783 10.1093/jac/47.5.675

[35] Riedel S, Neoh KM, Eisinger SW, Dam LM, Tekle T, Carroll KC. Comparison of commercial antimicrobial susceptibility test methods for testing of Staphylococcus aureus and Enterococci against vancomycin, daptomycin, and linezolid. J Clin Microbiol. 2014; 52: 2216–22. 10.1128/JCM.00957-14 24719445 PMC4042782

[36] Boncoeur E, Durmort C, Bernay B, Ebel C, Di Guilmi AM, Croizé J, et al PatA and PatB form a functional heterodimeric ABC multidrug efflux transporter responsible for the resistance of Streptococcus pneumoniae to fluoroquinolones. Biochemistry 2012; 51: 7755–7765. 10.1021/bi300762p 22950454

[37] Wang YC, Lipsitch M. Upgrading antibiotic use within a class: tradeoff between resistance and treatment success. Proc Natl Acad Sci U S A. 2006;103: 9655–9660. 16772381 10.1073/pnas.0600636103 PMC1480462

[38] Bowker KE, Garvey MI, Noel AR, Tomaselli SG, Macgowan AP. Comparative antibacterial effects of moxifloxacin and levofloxacin on Streptococcus pneumoniae strains with defined mechanisms of resistance: impact of bacterial inoculum. J Antimicrob Chemother. 2013; 68: 1130–8. 10.1093/jac/dks537 23361641

[39] Zhang G, Tian W, Wang C, Feng J. Identification of a novel resistance mutation in parE that confers high-level resistance to moxifloxacin in Streptococcus pneumoniae. J Antimicrob Chemother 2012; 67 (11): 2773–2774. 10.1093/jac/dks262 22761332

[40] Zhang G, Wang C, Sui Z, Feng J. Insights into the evolutionary trajectories of fluoroquinolone resistance in Streptococcus pneumoniae. J Antimicrob Chemother. 2015; 70: 2499–2506. 10.1093/jac/dkv134 26031465

[41] Guérin F, Galimand M, Tuambilangana F, Courvalin P, Cattoir V. Overexpression of the novel MATE fluoroquinolone efflux pump FepA in Listeria monocytogenes is driven by inactivation of its local repressor FepR. PLoS One 2014; 9: e106340 10.1371/journal.pone.0106340 25188450 PMC4154695

[42] Jacoby GA, Hooper DC. Phylogenetic analysis of chromosomally determined qnr and related proteins. Antimicrob Agents Chemother. 2013; 57: 1930–1934. 10.1128/AAC.02080-12 23318805 PMC3623327

[43] Tocci N, Iannelli F, Bidossi A, Ciusa ML, Decorosi F, Viti C, et al Functional analysis of pneumococcal drug efflux pumps associates the MATE DinF transporter with quinolone susceptibility. Antimicrob Agents Chemother. 2013; 57: 248–253. 10.1128/AAC.01298-12 23114782 PMC3535946

[44] Marrer E, Satoh AT, Johnson MM, Piddock LJ, Page MG. Global transcriptome analysis of the responses of a fluoroquinolone-resistant Streptococcus pneumoniae mutant and its parent to ciprofloxacin. Antimicrob Agents Chemother. 2006; 50: 269–278. 16377697 10.1128/AAC.50.1.269-278.2006 PMC1346767

