# Supplementary material for: Molecular Analysis of Rising Fluoroquinolone Resistance in Belgian Non-Invasive Streptococcus pneumoniae Isolates (1995-2014)
Source: PLoS One. 2016 May 26;11(5):e0154816. doi: 10.1371/journal.pone.0154816 (PMC4881901; doi:10.1371/journal.pone.0154816)
Supplement: S1 Fig — (DOCX) [file pone.0154816.s001.docx]

**
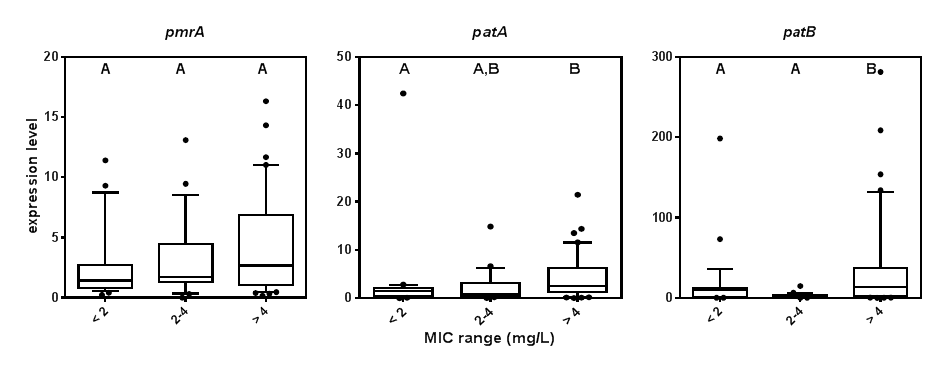
**

**S1 Figure. Gene expression analyses.** Boxplots showing the expression of *pmrA*, *patA* and *patB* in 94 tested strains, in comparison to the control strain *S. pneumoniae* ATCC 49619 and plotted in function of the MIC_CIP_. Plots with different letters indicate differences between groups that are statistically significant (p < 0.05) by the Kruskal-Wallis non-parametric test with Dunn's multiple test.
