# Supplementary material for: Molecular Analysis of Rising Fluoroquinolone Resistance in Belgian Non-Invasive Streptococcus pneumoniae Isolates (1995-2014)
Source: PLoS One. 2016 May 26;11(5):e0154816. doi: 10.1371/journal.pone.0154816 (PMC4881901; doi:10.1371/journal.pone.0154816)
Supplement: S1 Table — (DOCX) [file pone.0154816.s002.docx]

| **Strain** | **MIC distributions (µg/ml)** | | | |
| --- | --- | --- | --- | --- |
|  | Ofloxacin | Ciprofloxacin | Levofloxacin | Moxifloxacin |
| ***S*treptococcus pneumoniae**  **TPN 881** | 1.70±0.4 | 1.0±0.3 | 0.90±0.33 | 0.14±0.05 |
| ***Staphylococcus aureus* NCTC 11561** | 0.27±0.07 | 0.27±0.08 | 0.20±0.06 | 0.05±0.01 |
| ***Staphylococcus aureus* ATCC 29123** | 0.34±0.12 | 0.35±0.15 | 0.22±0.05 | 0.05±0.01 |

**S4 Table**. MIC distribution of the reference strains used in the broth microdilution experiments.
