# Supplementary material for: Molecular Analysis of Rising Fluoroquinolone Resistance in Belgian Non-Invasive Streptococcus pneumoniae Isolates (1995-2014)
Source: PLoS One. 2016 May 26;11(5):e0154816. doi: 10.1371/journal.pone.0154816 (PMC4881901; doi:10.1371/journal.pone.0154816)
Supplement: S2 Table — (DOCX) [file pone.0154816.s003.docx]

**S1 Table.** Oligonucleotides used in this study.

| **Name** | **Sequence (5’-3’)** | **Source** |
| --- | --- | --- |
| patA_up_F | GGCAGAAGAGCATCCTATCCTAG | This study |
| patA_up_R | CCAATCAACCAAGCCCCGATAC | This study |
| GyrA_F | CCTGTTCACCGTCGCATTCT | This study |
| GyrA_R | AGTTGCTCCATTAACCA | This study |
| GyrB_F | GTGCGCGTGAAGTCACACGTA | This study |
| GyrB_R | GCATCGGTCATCAAAACGAG | This study |
| ParC_F | CCGGGCTTTGCCAGATAT | This study |
| ParC_R | GGCTGCTGGCAAGACCGTT | This study |
| ParE_F | CAGCCCAATCTAAGAAT | This study |
| ParE_R | GCAATATAGACATGACCT | This study |
| rpoD-F | CAGGTAGCAGAATTTATCCGTAATC | PrimerDesign Ltd |
| rpoD-R | CCCATCAGCGTCCAAGGTA | PrimerDesign Ltd |
| proC-F | TTATCCCAAGTCAACACCGAAT | PrimerDesign Ltd |
| proC-R | GCAATTAGGAGACAAGGCATAAC | PrimerDesign Ltd |
| patB_R | AGGATATCGCCATCTTGTCG | [18] |
| patB_F | ATGGCAAAGCCTATCAGGAA | [18] |
| patA_F | TCCTGATGACAGGCTTGATG | [18] |
| patA_R | TGCGAGGACAACATTGAGTC | [18] |
| pmrA_F | TCCAGTATGGGCTTTTCCAG | [18] |
| pmrA_R | CCAATCCAAAGAGGAAACGA | [18] |
