# Supplementary material for: Molecular Analysis of Rising Fluoroquinolone Resistance in Belgian Non-Invasive Streptococcus pneumoniae Isolates (1995-2014)
Source: PLoS One. 2016 May 26;11(5):e0154816. doi: 10.1371/journal.pone.0154816 (PMC4881901; doi:10.1371/journal.pone.0154816)
Supplement: S3 Table — (DOCX) [file pone.0154816.s004.docx]

**S2 Table. Results of CST typing.** This is based on *wzh* sequences, which was chosen because it varies sufficiently between serotypes, but is conserved enough to amplify the same gene segment of the various serotypes using a single mix of primers. The scores are calculated as described by Elberse KE et al. (2011 PLoS One. 6:e20390).

| ID  (YEAR/ID) | Corresponding serotypes | Score |
| --- | --- | --- |
| ATCC | 19F | 99.7921 |
| 97a04 | 35F,47F | 99.799194 |
| 97b25 | 23F,15B,15C | 100.0 |
| 97B27 | 06A,06B,06C | 99.799194 |
| 99A16 | 23A | 99.393936 |
| 99G11 | 3 | 99.79253 |
| 99J16 | 11A,11D,18F | 99.8 |
| 01A38 | 23F,15B,15C | 99.799194 |
| 01C35 | 09V | 97.42063 |
| 01D01 | 06B | 100.0 |
| 01G34 | 11A,11D,18F | 100.0 |
| 01H21 | 23F,15B,15C | 99.59514 |
| 01H27 | 23F,15B,15C | 99.798386 |
| 01H28 | 23F,15B,15C | 99.799194 |
| 01J10 | 11A,11D,18F | 99.7996 |
| 03A07 | 09V | 99.5984 |
| 03B38f | 19A | 99.799194 |
| 03C18 | 09V | 99.79879 |
| 03K18 | 3 | 99.2016 |
| 03L23 | 06B,06A | 99.79798 |
| 03L28 | 31 | 99.799194 |
| 03N11 | 21 | 99.79879 |
| 04A24 | 4 | 99.5984 |
| 04J04 | 11A,11D,18F | 99.8004 |
| 05A20 | 20,13 | 94.69388 |
| 05A36 | 20,13 | 96.414345 |
| 05K36 | 22F,15B,22A | 99.799194 |
| 05M22 | 15A | 99.799194 |
| 06H02 | 09V | 97.95082 |
| 06H10 | 09N,09L | 99.79339 |
| 07A40 | 06B | 99.590164 |
| 07B16 | 3 | 99.2016 |
| 07H04 | 25F,25A,38 | 99.799194 |
| 07J30 | 25F,25A,38 | 99.799194 |
| 07O07 | 19F | 98.998 |
| 08E15 | 06A | 99.58506 |
| 08E16 | 22F,15B,22A | 99.794235 |
| 08L33 | 14 | 99.7921 |
| 09B07 | 19F | 98.998 |
| 09K33 | 19F | 99.40239 |
| 10D22 | 18C,18B, 19F | 94.02391 |
| 10D22 | 18C,18B, 19F | 94.03579 |
| 10K19 | 19A | 99.2 |
| 10N11 | 22F,15B,22A | 99.5842 |
| 11A23 | 15A | 99.198395 |
| 11A27 | 15A | 98.75776 |
| 13C24 | 07F,07A | 99.393936 |
| 13C28 | 09N,09L | 99.79296 |
| 13F15 | 35F,47F | 100.0 |
| 13G08 | 35F,47F | 99.79879 |
| 13J24 | 19A | 100.0 |
| 13K19 | 19F | 99.395164 |
| 13L04 | 23B | 100.0 |
