# Supplementary material for: Molecular Analysis of Rising Fluoroquinolone Resistance in Belgian Non-Invasive Streptococcus pneumoniae Isolates (1995-2014)
Source: PLoS One. 2016 May 26;11(5):e0154816. doi: 10.1371/journal.pone.0154816 (PMC4881901; doi:10.1371/journal.pone.0154816)
Supplement: S4 Table — (DOCX) [file pone.0154816.s005.docx]

**S3 Table.** QRDR sequencing and MIC determination of a selection of 422 pneumococcal strains.

| **Strain Id.** | **MIC (Microdilution)** | | | | **QRDR Sequencing** | | | | **MIC (E-tests)** | | | |
| --- | --- | --- | --- | --- | --- | --- | --- | --- | --- | --- | --- | --- |
|  | **CIP** | **LVX** | **MXF** | **OFL** | **GyrA** | **GyrB** | **ParC** | **ParE** | **CIP** | **CIP+R** | **MOX** | **MOX+R** |
| **95B01** | 4 | 2 | 0.25 | 4 | wt | wt | K137N | I460V | 1.5 | 1 | 0.19 | 0.19 |
| **95B03** | 4 | 2 | 0.25 | 4 | wt | wt | wt | I460V | 1 | 0.75 | 0.094 | 0.094 |
| **95B04** | 4 | 2 | 0.25 | 4 | wt | wt | wt | I460V | 0.75 | 0.75 | 0.125 | 0.125 |
| **95B05** | 2 | 2 | 0.12 | 2 | wt | wt | K137N | I460V | 0.38 | 0.19 | 0.047 | 0.047 |
| **95B08** | 4 | 2 | 0.25 | 4 | wt | wt | K137N | I460V | 1 | 0.75 | 0.125 | 0.125 |
| **95B09** | 4 | 2 | 0.25 | 4 | wt | wt | wt | wt | 1 | 0.75 | 0.19 | 0.19 |
| **95B11** | 4 | 2 | 0.25 | 4 | wt | wt | wt | I460V | 0.75 | 0.5 | 0.19 | 0.19 |
| **95B15** | 4 | 2 | 0.25 | 4 | S114G | wt | S52G | wt | 1.5 | 0.75 | 0.19 | 0.19 |
| **95B16** | 4 | 2 | 0.25 | 4 | S114G | wt | S52G/N91D | wt | 4 | 1 | 0.25 | 0.19 |
| **95B18** | 2 | 2 | 0.25 | 2 | wt | wt | wt | wt | 1.5 | 0.5 | 0.125 | 0.125 |
| **95B20** | 4 | 2 | 0.25 | 4 | M99I | wt | wt | I460V | 1.5 | 1 | 0.19 | 0.19 |
| **95B21** | 4 | 2 | 0.12 | 4 | S114G | wt | wt | K448P | 4 | 1 | 0.25 | 0.25 |
| **95C02** | 2 | 2 | 0.25 | 4 | wt | wt | wt | I460V | 0.75 | 0.5 | 0.125 | 0.125 |
| **95C08** | 4 | 2 | 0.25 | 4 | wt | wt | S52G/N91D | I460V | 1.5 | 0.5 | 0.19 | 0.19 |
| **95C12** | 4 | 2 | 0.25 | 4 | wt | wt | wt | I460V | 1.5 | 1.5 | 0.125 | 0.125 |
| **95C17** | 2 | 2 | 0.12 | 2 | wt | wt | wt | I460V | 0.75 | 0.25 | 0.094 | 0.094 |
| **95C20** | 2 | 2 | 0.12 | 2 | wt | wt | wt | wt | 0.5 | 0.38 | 0.125 | 0.125 |
| **95C24** | 2 | 2 | 0.12 | 2 | wt | wt | K137N | I460V | 1 | 0.5 | 0.125 | 0.125 |
| **95D06** | 2 | 2 | 0.12 | 2 | wt | wt | K137N | I460V | 0.5 | 0.38 | 0.125 | 0.094 |
| **95D08** | 2 | 1 | 0.12 | 2 | wt | wt | wt | I460V | 0.5 | 0.38 | 0.094 | 0.094 |
| **95D12** | 2 | 1 | 0.12 | 2 | wt | wt | K137N | wt | 0.75 | 0.38 | 0.125 | 0.094 |
| **95D19** | 2 | 1 | 0.12 | 2 | M99I | wt | wt | I460V | 0.75 | 0.38 | 0.094 | 0.094 |
| **95E02** | 2 | 2 | 0.25 | 4 | wt | wt | wt | I460V | 1 | 0.5 | 0.125 | 0.125 |
| **95E11** | 2 | 1 | 0.12 | 2 | wt | wt | wt | wt | 1 | 0.5 | 0.125 | 0.125 |
| **95F03** | 2 | 1 | 0.25 | 2 | G103S | wt | wt | I460V | 0.75 | 0.5 | 0.125 | 0.125 |
| **95F08** | 2 | 1 | 0.12 | 2 | wt | wt | wt | I460V | 2 | 0.5 | 0.094 | 0.094 |
| **95G05** | 2 | 1 | 0.12 | 2 | wt | wt | wt | I460V | 0.75 | 0.5 | 0.125 | 0.125 |
| **95G06** | 2 | 1 | 0.12 | 2 | wt | wt | wt | I460V | 1 | 0.5 | 0.125 | 0.094 |
| **95H07** | 2 | 1 | 0.06 | 4 | wt | wt | K137N | I460V | 0.38 | 0.19 | 0.064 | 0.064 |
| **95H10** | 2 | 1 | 0.12 | 4 | wt | wt | wt | I460V | 0.75 | 0.38 | 0.125 | 0.125 |
| **97A04** | 8 | 4 | 0.25 | 4 | wt | wt | S79F | I460V | 8 | 3 | 0.25 | 0.25 |
| **97A11** | 2 | 1 | 0.25 | 4 | wt | wt | wt | I460V | 2 | 0.5 | 0.125 | 0.125 |
| **97B03** | 2 | 1 | 0.06 | 2 | wt | wt | wt | I460V | 1 | 0.38 | 0.094 | 0.064 |
| **97B14** | 2 | 2 | 0.12 | 4 | L154F | wt | wt | I460V | 1.5 | 0.5 | 0.094 | 0.094 |
| **97B15** | 2 | 2 | 0.25 | 4 | wt | wt | wt | I460V | 0.75 | 0.5 | 0.094 | 0.094 |
| **97B18** | 2 | 2 | 0.12 | 4 | wt | wt | wt | I460V | 0.5 | 0.38 | 0.064 | 0.064 |
| **97B21** | 2 | 1 | 0.12 | 4 | wt | wt | wt | wt | 2 | 0.38 | 0.125 | 0.094 |
| **97B23** | 4 | 2 | 0.12 | 4 | wt | wt | wt | I460V | 1 | 0.75 | 0.19 | 0.19 |
| **97B25** | 4 | 4 | 0.25 | 8 | wt | wt | ND | wt | 24 | 4 | 0.38 | 0.38 |
| **97B27** | 2 | 2 | 0.12 | 4 | wt | wt | wt | I460V | 8 | 0.75 | 0.094 | 0.094 |
| **97C08** | 2 | 2 | 0.12 | 8 | wt |  | wt | I460V | 1 | 0.75 | 0.19 | 0.19 |
| **97C12** | 2 | 2 | 0.12 | 4 | wt | wt | wt | I460V | 1.5 | 0.5 | 0.125 | 0.125 |
| **97C13** | 2 | 1 | 0.06 | 4 | wt | wt | wt | I460V | 1 | 0.5 | 0.125 | 0.125 |
| **97C16** | 2 | 1 | 0.12 | 4 | wt | wt | wt | wt | 2 | 0.38 | 0.094 | 0.094 |
| **97C24** | 2 | 1 | 0.12 | 4 | wt | wt | wt | wt | 0.5 | 0.38 | 0.094 | 0.094 |
| **97D04** | 2 | 2 | 0.25 | 4 | wt | wt | K137N | I460V | 1 | 0.38 | 0.094 | 0.094 |
| **97D22** | 2 | 0.5 | 0.06 | 2 | wt | wt | wt | I460V | 1 | 0.5 | 0.094 | 0.094 |
| **97G03** | 2 | 0.5 | 0.06 | 2 | wt | wt | S79F | A532T | 2 | 1 | 0.19 | 0.125 |
| **97G09** | 2 | 1 | 0.12 | 2 | wt | wt | S79F | wt | 4 | 1.5 | 0.125 | 0.125 |
| **97H12** | 2 | 1 | 0.12 | 2 | wt | wt | wt | wt | 0.75 | 0.38 | 0.064 | 0.064 |
| **97H26** | 2 | 1 | 0.12 | 2 | wt | wt | K137N | I460V | 0.5 | 0.38 | 0.094 | 0.094 |
| **97I27** | 2 | 1 | 0.12 | 2 | wt | wt | wt | I460V | 1.5 | 0.5 | 0.125 | 0.094 |
| **99A01** | 0.5 | 0.06 | 0.5 | 0.06 | wt | wt | K137N | I460V | 0.5 | 0.25 | 0.094 | 0.064 |
| **99A05** | 2 | 2 | 0.12 | 4 | wt | wt | wt | wt | 1.5 | 0.5 | 0.125 | 0.125 |
| **99A09** | 2 | 0.5 | 2 | 0.5 | wt | wt | wt | I460V | 2 | 0.5 | 0.19 | 0.125 |
| **99A11** | 2 | 2 | 0.12 | 4 | wt | wt | wt | wt | 1.5 | 0.75 | 0.125 | 0.125 |
| **99A12** | 2 | 2 | 0.06 | 4 | wt | wt | K137N | I460V | 0.75 | 0.38 | 0.094 | 0.094 |
| **99A16** | 4 | 4 | 4 | 0.5 | S81F | wt | D83G | wt | 32 | 16 | 2 | 1.5 |
| **99B11** | 1 | 0.25 | 1 | 0.12 | wt | wt | wt | I460V | 1 | 0.38 | 0.125 | 0.094 |
| **99C21** | 2 | 2 | 0.25 | 4 | wt | wt | wt | I460V | 1 | 0.38 | 0.094 | 0.094 |
| **99C23** | 4 | 1 | 4 | 0.25 | S114G | wt | S52G/N91D/E134D | opnieuw | 0.75 | 0.38 | 0.125 | 0.094 |
| **99C25** | 1 | 0.5 | 2 | 0.25 | wt | wt | wt | I460V | 1.5 | 0.38 | 0.094 | 0.094 |
| **99D02** | 2 | 0.12 | 2 | 0.06 | wt | wt | K137N | I460V | 4 | 0.75 | 0.094 | 0.064 |
| **99D21** | 2 | 2 | 0.06 | 4 | wt | wt | N91D | I460V | 0.75 | 0.25 | 0.064 | 0.064 |
| **99D22** | 2 | 2 | 0.12 | 4 | wt | wt | wt | wt | 0.75 | 0.38 | 0.064 | 0.064 |
| **99D25** | 2 | 0.5 | 2 | 0.25 | wt | wt | K137N | I460V | 1 | 0.5 | 0.125 | 0.094 |
| **99E12** | 2 | 1 | 2 | 0.25 | wt | wt | K137N | I460V | 1 | 0.5 | 0.094 | 0.094 |
| **99 E22** | 2 | 2 | 0.25 | 4 | wt | wt | wt | I460V | 1 | 0.5 | 0.094 | 0.094 |
| **99 E24** | 2 | 2 | 0.12 | 4 | wt | wt | wt | I460V | 4 | 1 | 0.094 | 0.094 |
| **99G09** | 2 | 2 | 0.5 | 4 | wt | wt | wt | I460V | 1.5 | 0.5 | 0.125 | 0.125 |
| **99G10** | 2 | 2 | 0.25 | 4 | wt | wt | wt | I460V | 2 | 0.5 | 0.125 | 0.125 |
| **99G11** | 2 | 2 | 0.5 | 4 | wt | wt | wt | I460V | 6 | 0.75 | 0.125 | 0.125 |
| **99G16** | 2 | 2 | 0.5 | 4 | wt | wt | wt | I460V | 0.5 | 0.38 | 0.094 | 0.094 |
| **99G17** | 2 | 0.5 | 2 | 0.25 | wt | wt | K137N | I460V | 1 | 0.5 | 0.125 | 0.125 |
| **99G21** | 2 | 0.5 | 2 | 0.25 | wt | wt | wt | I460V | 1 | 0.5 | 0.125 | 0.125 |
| **99G24** | 4 | 2 | 0.5 | 8 | wt | wt | wt | I460V | 2 | 0.5 | 0.125 | 0.125 |
| **99H10** | 2 | 0.25 | 2 | 0.12 | wt | wt | wt | wt | 1.5 | 0.19 | 0.094 | 0.094 |
| **99H17** | 2 | 0.5 | 2 | 0.25 | wt | wt | wt | wt | 0.5 | 0.125 | 0.064 | 0.032 |
| **99H18** | 2 | 1 | 2 | 0.5 | wt | wt | wt | I460V | 1.5 | 1 | 0.19 | 0.125 |
| **99I23** | 2 | 0.5 | 2 | 0.25 | wt | wt | wt | I460V | 1 | 0.38 | 0.125 | 0.125 |
| **99I24** | 2 | 2 | 0.12 | 4 | L155F | wt | wt | I460V | 0.5 | 0.38 | 0.094 | 0.094 |
| **99J07** | 2 | 2 | 0.25 | 4 | wt | wt | K137N | I460V | 0.75 | 0.5 | 0.125 | 0.125 |
| **99J08** | 2 | 1 | 0.12 | 4 | wt | wt | wt | I460V | 4 | 0.75 | 0.125 | 0.125 |
| **99J13** | 2 | 0.5 | 2 | 0.25 | wt | wt | wt | I460V | 0.75 | 0.38 | 0.094 | 0.094 |
| **99J16** | 4 | 1 | 4 | 0.5 | wt | wt | wt | I460V | 8 | 0.38 | 0.094 | 0.094 |
| **99J20** | 2 | 1 | 4 | 0.5 | wt | wt | K137N | I460V | 1 | 0.5 | 0.125 | 0.125 |
| **01A01** | 2 | 1 | 0.25 | 4 | wt | wt | wt | Q420P/I460V | 1 | 0.38 | 0.125 | 0.125 |
| **01A05** | 2 | 1 | 0.25 | 4 | wt | wt | wt | I460V | 1 | 0.5 | 0.125 | 0.125 |
| **01A07** | 2 | 2 | 0.25 | 4 | wt | wt | wt | I460V | 1 | 0.5 | 0.094 | 0.094 |
| **01A09** | 4 | 2 | 0.12 | 8 | wt | wt | wt | wt | 0.75 | 0.5 | 0.125 | 0.094 |
| **01A38** | 4 | 2 | 0.12 | 8 | wt | wt | K137N | I460V | 12 | 2 | 0.25 | 0.25 |
| **01C01** | 4 | 4 | 0.5 | 8 | wt | wt | wt | I460V | 1 | 0.75 | 0.125 | 0.125 |
| **01C02** | 4 | 4 | 0.5 | 8 | wt | wt | wt | I460V/ D435N | 0.75 | 0.75 | 0.125 | 0.19 |
| **01C03** | 4 | 2 | 0.5 | 8 | S114G | wt | wt | H534L | 1 | 0.25 | 0.064 | 0.094 |
| **01C06** | 4 | 2 | 0.5 | 8 | wt | wt | K137N | I460V | 3 | 0.38 | 0.125 | 0.125 |
| **01C24** | 1 |  |  |  | S114G | wt | wt | I460V | 0.75 | 0.25 | 0.094 | 0.094 |
| **01C30** | 2 | 2 | 0.25 | 4 | wt | wt | K137N | I460V | 1 | 0.38 | 0.094 | 0.094 |
| **01C33** | 2 | 2 | 0.25 | 4 | wt | wt | wt | I460V | 1 | 0.5 | 0.125 | 0.125 |
| **01C35** | 4 | 4 | 0.25 | 8 | S114G | wt | S79Y/N91D/E213D | I460V | 16 | 2 | 0.125 | 0.125 |
| **01D01** | 2 | 2 | 0.25 | 4 | wt | wt | K137N | wt | 6 | 0.75 | 0.125 | 0.125 |
| **01D02** | 2 | 2 | 0.12 | 4 | wt | wt | wt | I460V | 0.75 | 0.5 | 0.125 | 0.094 |
| **01D04** | 2 | 2 | 0.12 | 4 | wt | wt | wt | I460V | 0.5 | 0.5 | 0.125 | 0.125 |
| **01D33** | 2 | 2 | 0.25 | 4 | wt | wt | wt | I460V | 0.75 | 0.5 | 0.125 | 0.125 |
| **01F05** | 2 | 2 | 0.12 | 4 | wt | wt | wt | I460V | 0.5 | 0.38 | 0.094 | 0.064 |
| **01G07** | 2 | 2 | 0.25 | 4 | wt | wt | wt | wt | 0.75 | 0.38 | 0.094 | 0.094 |
| **01G18** | 2 | 2 | 0.12 | 4 | wt | wt | wt | I460V | 4 | 0.38 | 0.125 | 0.094 |
| **01G34** | 8 | 4 | 0.25 | 8 | wt | wt | D83Y | I460V | 12 | 1 | 0.125 | 0.125 |
| **01H12** | 2 | 2 | 0.25 | 4 | wt | wt | N91D / E135D | I460V | 1 | 0.5 | 0.125 | 0.125 |
| **01H21** | 4 | 4 | 1 | 8 | S81F | wt | K137N | I460V/ D435N | 16 | 4 | 1.5 | 1 |
| **01H27** | 4 | 4 | 0.5 | 8 | S81F | wt | K137N | I460V/ D435N | 32 | 12 | 3 | 2 |
| **01H28** | 4 | 4 | 1 | 8 | S81F | wt | K137N | I460V | >32 | 12 | 3 | 2 |
| **01I04** | 2 | 2 | 0.12 | 4 | wt | wt | wt | I460V | 1 | 0.5 | 0.094 | 0.094 |
| **01J06** | 2 | 2 | 0.25 | 4 | wt | wt | K137N | I460V | 1 | 0.5 | 0.125 | 0.125 |
| **01J10** | 8 | 8 | 2 | 16 | S81F | wt | S79F | I460V | 32 | 32 | 2 | 3 |
| **01J38** | 2 | 1 | 0.06 | 4 | wt | wt | wt | I460V | 4 | 1 | 0.125 | 0.125 |
| **03 A05** | 2 | 0.5 | 2 | 4 | wt | wt | wt | I460V | 1.5 | 1 | 0.125 | 0.125 |
| **03 A07** | 8 | 8 | 8 | 8 | S81Y/ S114G | wt | S79Y/N91D/E134D | ND | 32 | 32 | 4 | 4 |
| **03 A22** | 2 | 0.12 | 1 | 4 | S114G | wt | wt | I460V | 1.5 | 0.5 | 0.094 | 0.094 |
| **03 B29** | 2 | 0.25 | 1 | 4 | wt | wt | wt | I460V | 0.75 | 0.75 | 0.094 | 0.094 |
| **03B38** | 2 | 1 | 0.25 | 4 | wt | wt | S79Y | I460V | 8 | 2 | 0.125 | 0.125 |
| **03 C09** | 2 | 0.25 | 2 | 4 | M99I | wt | wt | I460V | 0.75 | 0.75 | 0.094 | 0.125 |
| **03 C10** | 2 | 0.12 | 1 |  | wt | wt | wt | I460V | 2 | 0.75 | 0.064 | 0.125 |
| **03 C14** | 2 | 0.12 | 2 | 4 | wt | wt | wt | wt | 3 | 0.75 | 0.094 | 0.125 |
| **03 C18** | 8 | 2 | 8 | 16 | E85K | wt | S79Y/K137N | I460V | 32 | 32 | 3 | 4 |
| **03 D03** | 2 | 0.12 | 2 | 4 | wt | wt | wt | I460V | 0.75 | 0.5 | 0.064 | 0.064 |
| **03 D06** | 4 | 0.25 | 4 | 8 | S114G/ F161L | wt | N91D/S52G | wt | 1 | 0.75 | 0.094 | 0.125 |
| **03 D11** | 2 | 0.12 | 2 | 4 | wt | wt | wt | I460V | 0.75 | 0.75 | 0.047 | 0.094 |
| **03 D13** | 2 | 0.25 | 1 | 4 | wt | wt | wt | I460V | 1.5 | 0.75 | 0.094 | 0.094 |
| **03 D17** | 2 | 0.12 | 1 | 4 | wt | wt | wt | I460V | 1 | 0.5 | 0.064 | 0.064 |
| **03 D27** | 2 | 0.06 | 1 | 4 | wt | wt | wt | I460V | 0.75 | 0.5 | 0.094 | 0.094 |
| **03 E04** | 2 | 0.12 | 1 | 4 | wt | wt | wt | wt | 0.75 | 0.5 | 0.094 | 0.125 |
| **03 E10** | 4 | 0.12 | 2 | 8 | wt | wt | wt | I460V | 1 | 0.75 | 0.094 | 0.094 |
| **03 E11** | 8 | 0.25 | 8 | 32 | wt | wt | wt | I460V | 0.5 | 0.38 | 0.047 | 0.094 |
| **03 E12** | 4 | 0.25 | 4 | 8 | wt | wt | wt | I460V | 1 | 0.75 | 0.094 | 0.125 |
| **03 E13** | 4 | 0.12 | 2 | 8 | wt | wt | wt | ND | 0.75 | 0.75 | 0.064 | 0.064 |
| **03 E14** | 2 | 0.06 | 2 | 4 | wt | wt | wt | I460V | 1.5 | 0.38 | 0.094 | 0.094 |
| **03 E15** | 4 | 0.12 | 4 | 8 | wt | wt | wt | I460V | 0.38 | 0.38 | 0.047 | 0.094 |
| **03 E17** | 8 | 0.12 | 8 | 16 | wt | wt | wt | wt | 2 | 0.75 | 0.094 | 0.125 |
| **03 E18** | 8 | 0.25 | 8 | 32 | wt | wt | S79F/K137N | I460V | 3 | 1.5 | 0.125 | 0.19 |
| **03 E19** | 8 | 0.12 | 8 | 16 | S114G/ L152R | wt | S52G/K57Q/N91D | wt | 2 | 0.5 | 0.094 | 0.125 |
| **03 E21** | 4 | 0.12 | 4 | 8 | wt | wt | wt | wt | 0.75 | 0.75 | 0.094 | 0.125 |
| **03 G16** | 2 | 0.12 | 2 | 4 | wt | wt | wt | wt | 2 | 0.75 | 0.125 | 0.125 |
| **03 H10** | 2 | 0.12 | 2 |  | wt | wt | wt | I460V | 2 | 0.5 | 0.125 | 0.094 |
| **03H12** | 4 | 0.12 | 4 | 8 | wt | wt | wt | I460V | 0.5 | 0.5 | 0.064 | 0.064 |
| **03 H32** | 4 | 0.12 | 4 | 8 | S114G/L155V | wt | S52G/N91D | Y481H/ I493L | 1.5 | 0.75 | 0.094 | 0.125 |
| **03 I06** | 2 | 0.12 | 1 | 4 | wt | wt | wt | I460V | 2 | 1 | 0.125 | 0.125 |
| **03 I08** | 2 | 0.5 | 1 | 4 | wt | wt | wt | I460V | 1 | 0.5 | 0.094 | 0.094 |
| **03 I12** | 2 | 0.25 | 1 | 4 | wt | wt | wt | I460V | 2 | 0.75 | 0.125 | 0.125 |
| **03 I21** | 2 | 0.12 | 2 | 4 | wt | wt | wt | I460V | 1.5 | 0.75 | 0.125 | 0.125 |
| **03 I23** | 2 | 0.25 | 2 | 4 | wt | wt | wt | I460V | 2 | 0.75 | 0.125 | 0.125 |
| **03 J25** | 2 | 0.12 | 1 | 4 | wt | wt | wt | I460V | 1 | 0.38 | 0.094 | 0.064 |
| **03 K18** | 4 | 0.12 | 2 | 8 | wt | wt | wt | I460V | 8 | 0.75 | 0.125 | 0.125 |
| **03K32** | 2 | 2 | 0.25 | 4 | wt | wt | S79F | wt | 2 | 1.5 | 0.125 | 0.125 |
| **03K33** | 2 | 2 | 0.25 | 4 | wt | wt | K137N | I460V | 0.75 | 0.75 | 0.125 | 0.125 |
| **03K36** | 2 | 2 | 0.5 | 4 | wt | wt | wt | I460V | 1 | 0.5 | 0.064 | 0.125 |
| **03K38** | 2 | 1 | 0.25 | 4 | wt | wt | wt | wt | 2 | 0.5 | 0.125 | 0.125 |
| **03L03** | 2 | 2 | 0.25 | 4 | wt | wt | wt | I460V | 0.5 | 0.5 | 0.094 | 0.064 |
| **03L14** | 2 | 1 | 0.25 | 4 | wt | wt | wt | I460V | 2 | 0.5 | 0.094 | 0.094 |
| **03L15** | 2 | 2 | 0.25 | 4 | wt | wt | wt | I460V | 0.5 | 0.5 | 0.125 | 0.125 |
| **03 L23** | 8 | 0.12 | 4 | 8 | wt | wt | S79F | D435K/H534L | 32 | 2 | 0.19 | 0.19 |
| **03L28** | 2 | 1 | 0.12 | 4 | wt | wt | K137N | I460V | >32 | 1.5 | 0.125 | 0.125 |
| **03L31** | 2 | 1 | 0.12 | 4 | wt | F480L | wt | wt | 0.5 | 0.25 | 0.064 | 0.064 |
| **03N04** | 2 | 1 | 0.12 | 2 | wt | wt | K137N | I460V | 1.5 | 0.75 | 0.125 | 0.125 |
| **03N06** | 2 | 1 | 0.12 | 4 | wt | wt | K137N | I460V | 1.5 | 0.75 | 0.125 | 0.064 |
| **03N11** | 2 | 1 | 0.12 | 4 | wt | wt | S79Y | I460V | 16 | 2 | 0.19 | 0.19 |
| **03 O03** | 2 | 0.12 | 2 | 4 | wt | wt | S52G/N91D/R95G | I460V | 1 | 0.75 | 0.94 | 0.94 |
| **03 O06** | 4 | 0.12 | 2 | 8 | wt | wt | wt | I460V | 0.5 | 0.5 | 0.094 | 0.125 |
| **03O10** | 2 | 1 | 0.12 | 4 | wt | wt | wt | I460V | 1 | 0.75 | 0.125 | 0.125 |
| **03O22** | 2 | 1 | 0.25 | 4 | wt | wt | wt | I460V | 3 | 1 | 0.19 | 0.19 |
| **04A06** | 2 | 2 | 0.25 | 4 | wt | wt | K137N | I460V | 0.75 | 0.5 | 0.125 | 0.125 |
| **04A07** | 2 | 2 | 0.25 | 4 | wt | wt | wt | I460V | 0.5 | 0.5 | 0.125 | 0.125 |
| **04A10** | 2 | 2 | 0.25 | 4 | wt | wt | wt | I460V | 1.5 | 0.5 | 0.125 | 0.125 |
| **04A24** | 2 | 2 | 0.25 | 4 | wt | wt | wt | I460V | 8 | 1 | 0.19 | 0.125 |
| **04A25** | 2 | 2 | 0.12 | 4 | wt | wt | wt | I460V | 3 | 1 | 0.19 | 0.19 |
| **04A30** | 2 | 2 | 0.12 | 4 | S114G | wt | K137N | I460V | 1 | 0.38 | 0.125 | 0.125 |
| **04B04** | 4 | 4 | 0.25 | 8 | S114G | F491I/S494T | wt | I460V | 0.75 | 0.38 | 0.064 | 0.064 |
| **04B11** | 4 | 4 | 0.25 | 8 | wt | wt | K137N | I460V | 0.5 | 0.25 | 0.094 | 0.064 |
| **04B15** | 2 | 2 | 0.06 | 4 |  |  |  |  |  |  |  |  |
| **04B16** | 4 | 4 | 0.25 | 8 | wt | wt | wt | I460V | 1.5 | 0.75 | 0.125 | 0.125 |
| **04C03** | 4 | 4 | 0.06 | 8 | wt | wt | wt | I460V | 0.38 | 0.38 | 0.064 | 0.064 |
| **04C04** | 4 | 4 | 0.12 | 8 | wt | wt | wt | I460V | 1.5 | 0.5 | 0.094 | 0.094 |
| **04D19** | 2 | 2 | 0.12 | 4 | wt | wt | wt | I460V | 1 | 0.5 | 0.125 | 0.094 |
| **04E02** | 4 | 4 | 0.25 | 8 | wt | wt | wt | I460V | 0.75 | 0.5 | 0.125 | 0.125 |
| **04E05** | 2 | 4 | 0.25 | 4 | wt | wt | wt | wt | 0.5 | 0.38 | 0.094 | 0.094 |
| **04E10** | 2 | 4 | 0.25 | 4 | wt | wt | wt | I460V | 1.5 | 0.5 | 0.125 | 0.125 |
| **04F27** | 4 | 4 | 0.12 | 8 | wt | wt | wt | I460V | 1 | 0.38 | 0.094 | 0.064 |
| **04H30** | 2 | 2 | 0.12 | 4 | wt | wt | wt | I460V | 1 | 0.38 | 0.094 | 0.094 |
| **04H32** | 2 | 4 | 0.12 | 4 | wt | wt | wt | A532V | 1 | 0.38 | 0.094 | 0.094 |
| **04I06** | 2 | 2 | 0.12 | 4 |  |  |  |  |  |  |  |  |
| **04I29** | 2 | 1 | 0.06 | 4 | wt | wt | wt | I460V | 1 | 0.5 | 0.094 | 0.094 |
| **04I40** | 2 | 1 | 0.12 | 4 | wt | wt | wt | I460V | 0.5 | 0.38 | 0.125 | 0.125 |
| **04J04** | 16 | 16 | 2 | 16 | S81Y | wt | S79F | I460V | >32 | >32 | 1 | 1 |
| **04J07** | 2 | 1 | 0.06 | 4 | wt | wt | K137N | I460V | 0.38 | 0.25 | 0.064 | 0.064 |
| **04J15** | 2 | 2 | 0.06 | 4 | wt | wt | wt | I460V | 0.75 | 0.38 | 0.094 | 0.094 |
| **04J18** | 2 | 2 | 0.12 | 4 | wt | wt | wt | I460V | 1 | 0.5 | 0.125 | 0.125 |
| **04K11** | 4 | 2 | 0.12 | 8 | wt | wt | wt | I460V | 1 | 0.38 | 0.094 | 0.094 |
| **04L02** | 2 | 1 | 0.12 | 4 | wt | wt | wt | I460V | 0.75 | 0.5 | 0.094 | 0.094 |
| **04L03** | 2 | 1 | 0.12 | 4 | wt | wt | wt | I460V | 0.75 | 0.38 | 0.094 | 0.094 |
| **04L06** | 2 | 2 | 0.25 | 4 | wt | wt | wt | wt | 0.5 | 0.5 | 0.094 | 0.094 |
| **04L07** | 2 | 2 | 0.12 | 4 | wt | wt | wt | I460V | 1.5 | 0.5 | 0.094 | 0.094 |
| **04L17** | 4 | 4 | 0.5 | 8 | S81F | wt | K137N | I460V | 2 | 1 | 0.5 | 0.38 |
| **04L24** | 2 | 2 | 0.12 | 4 | wt | wt | K137N | I460V | 1.5 | 0.5 | 0.094 | 0.094 |
| **04L31** | 2 | 2 | 0.12 | 4 | wt | wt | wt | I460V | 2 | 0.75 | 0.125 | 0.125 |
| **04M10** | 2 | 2 | 0.12 | 4 | wt | wt | wt | I460V | 0.38 | 0.25 | 0.064 | 0.064 |
| **04N12** | 2 | 2 | 0.06 | 4 | wt | wt | wt | I460V | 0.5 | 0.38 | 0.094 | 0.094 |
| **04N39** | 2 | 2 | 0.12 | 4 | wt | wt | K137N | I460V | 0.5 | 0.5 | 0.094 | 0.094 |
| **04O22** | 2 | 2 | 0.12 | 4 | wt | wt | wt | I460V | 0.5 | 0.38 | 0.094 | 0.094 |
| **05A02** | 2 | 2 | 0.25 | 4 | wt | wt | K137N | I460V | 0.75 | 0.38 | 0.094 | 0.094 |
| **05A05** | 2 | 2 | 0.25 | 4 | S81F | wt | K57T | I460V | 1 | 0.5 | 0.125 | 0.094 |
| **05A07** | 2 | 2 | 0.25 | 4 | wt | S466G | K57T | I460V | 0.75 | 0.5 | 0.125 | 0.094 |
| **05A13** | 4 | 2 | 0.25 | 8 | S114I | wt | K57T | I460V | 1.5 | 0.5 | 0.125 | 0.125 |
| **05A15** | 2 | 2 | 0.25 | 4 | wt | wt | K57T | I460V | 1.5 | 0.75 | 0.125 | 0.125 |
| **05A20** | 64 | 64 | 64 | 64 | S81L\ S114G | P454S | wt | wt | >32 | >32 | >32 | >32 |
| **05A28** | 2 | 2 | 0.25 | 4 | S114G | wt | S52G/N91D/T54N/K57T | wt | 4 | 1 | 0.19 | 0.19 |
| **05A34** | 2 | 2 | 0.25 | 4 | S114G | wt | S52G/N91D/K57L/ | wt | 4 | 0.75 | 0.19 | 0.19 |
| **05A36** | 8 | 2 | 0.25 | 8 | wt | wt | S79Y/K137N/K57T | wt | 16 | 3 | 0.25 | 0.19 |
| **05B29** | 2 | 2 | 0.25 | 4 | wt | wt | K57T | I460V | 1 | 0.38 | 0.125 | 0.125 |
| **05C03** | 2 | 1 | 0.25 | 4 | wt | wt | K57T | I460V | 1.5 | 0.75 | 0.125 | 0.125 |
| **05C07** | 2 | 1 | 0.12 | 4 | wt | wt | K137N/K57M | I460V | 0.75 | 0.5 | 0.125 | 0.125 |
| **05C32** | 2 | 1 | 0.12 | 4 | wt | wt | wt | I460V | 1.5 | 0.5 | 0.064 | 0.047 |
| **05C40** | 2 | 1 | 0.06 | 4 | wt | wt | K137N | I460V | 1 | 0.38 | 0.125 | 0.094 |
| **05D25** | 2 | 2 | 0.25 | 4 | wt | wt | wt | I460V | 1 | 0.5 | 0.125 | 0.125 |
| **05D26** | 2 | 2 | 0.12 | 4 | wt | wt | wt | I460V/ l431S | 1 | 0.75 | 0.125 | 0.125 |
| **05D28** | 2 | 2 | 0.12 | 4 | wt | wt | wt | I460V | 1 | 0.5 | 0.094 | 0.094 |
| **05D30** | 2 | 2 | 0.12 | 4 | wt | wt | wt | I460V | 1 | 0.5 | 0.125 | 0.125 |
| **05D31** | 2 | 2 | 0.25 | 4 | wt | wt | wt | I460V | 1.5 | 0.5 | 0.094 | 0.094 |
| **05D32** | 2 | 2 | 0.5 | 4 | wt | wt | wt | I460V | 1 | 0.5 | 0.094 | 0.094 |
| **05D34** | 4 | 2 | 0.25 | 8 | wt | wt | wt | I460V | 4 | 1 | 0.125 | 0.125 |
| **05D36** | 4 | 4 | 0.5 | 8 | wt | wt | wt | I460V | 1.5 | 0.5 | 0.125 | 0.125 |
| **05D39** | 2 | 2 | 0.25 | 4 | wt | wt | wt | I460V | 0.5 | 0.5 | 0.094 | 0.094 |
| **05D40** | 4 | 4 | 0.5 | 8 | wt | wt | wt | I460V | 1.5 | 0.5 | 0.125 | 0.125 |
| **05E02** | 2 | 2 | 0.25 | 4 | wt | wt | wt | I460V | 0.75 | 0.5 | 0.125 | 0.125 |
| **05E04** | 2 | 2 | 0.25 | 4 | wt | wt | wt | I460V | 1 | 0.5 | 0.125 | 0.125 |
| **05E10** | 2 | 1 | 0.12 | 4 | wt | wt | K137N | wt | 1.5 | 0.5 | 0.125 | 0.125 |
| **05E30** | 2 | 2 | 0.25 | 4 | wt | wt | wt | I460V | 1 | 0.5 | 0.125 | 0.125 |
| **05E36** | 2 | 2 | 0.25 | 4 | wt | wt | D78N | I460V | 2 | 1 | 0.19 | 0.19 |
| **05F15** | 2 | 1 | 0.12 | 4 | wt | wt | wt | I460V | 1 | 0.5 | 0.125 | 0.125 |
| **05I37** | 2 | 2 | 0.12 | 4 | wt | wt | wt | I460V | 0.75 | 0.5 | 0.19 | 0.19 |
| **05J31** | 2 | 2 | 0.25 | 4 | wt | wt | wt | I460V | 0.75 | 0.38 | 0.094 | 0.094 |
| **05K34** | 2 | 2 | 0.25 | 4 | wt | wt | S79F | wt | 4 | 1.5 | 0.19 | 0.19 |
| **05K36** | 16 | 16 | 2 | 16 | S81F | wt | S79F | wt | >32 | 32 | 4 | 3 |
| **05M22** | 2 | 1 | 0.12 | 4 | wt | wt | S79F | I460V | 6 | 2 | 0.19 | 0.19 |
| **06A02** | 2 | 1 | 0.12 | 4 | wt | wt | wt | I460V | 0.5 | 0.5 | 0.064 | 0.094 |
| **06A03** | 2 | 2 | 0.12 | 4 | wt | wt | wt | I460V | 1 | 0.5 | 0.125 | 0.125 |
| **06A05** | 2 | 1 | 0.12 | 4 | wt | wt | wt | wt | 1 | 0.5 | 0.125 | 0.125 |
| **06A07** | 2 | 2 | 0.12 | 2 | wt | wt | K137N | I460V | 0.75 | 0.38 | 0.094 | 0.094 |
| **06A08** | 2 | 2 | 0.12 | 4 | S114G | wt | wt | M467I/ A496T | 2 | 0.5 | 0.094 | 0.125 |
| **06A10** | 2 | 2 | 0.12 | 4 | wt | wt | wt | I460V | 1 | 0.5 | 0.125 | 0.125 |
| **06A11** | 2 | 1 | 0.25 | 4 | wt | wt | wt | wt | 0.5 | 0.5 | 0.125 | 0.094 |
| **06A12** | 2 | 1 | 0.12 | 4 | wt | wt | K137N | I460V | 0.75 | 0.5 | 0.064 | 0.094 |
| **06A13** | 2 | 2 | 0.25 | 4 | wt | wt | K137N | I460V | 0.5 | 0.5 | 0.125 | 0.094 |
| **06A15** | 2 | 2 | 0.12 | 4 | wt | wt | wt | I460V | 2 | 1 | 0.125 | 0.125 |
| **06A19** | 2 | 1 | 0.12 | 4 | wt | wt | wt | I460V | 0.75 | 0.5 | 0.094 | 0.125 |
| **06A20** | 2 | 2 | 0.12 | 4 | wt | wt | wt | I460V | 1.5 | 1 | 0.5 | 0.5 |
| **06A22** | 2 | 1 | 0.12 | 4 | wt | wt | wt | I460V | 0.75 | 0.38 | 0.125 | 0.125 |
| **06A24** | 2 | 2 | 0.12 | 4 | wt | wt | wt | I460V | 1.5 | 0.5 | 0.125 | 0.125 |
| **06A25** | 2 | 2 | 0.12 | 4 | wt | wt | wt | I460V | 1 | 0.5 | 0.094 | 0.125 |
| **06B18** | 2 | 2 | 0.25 | 4 | wt | wt | wt | I460V | 0.75 | 0.38 | 0.094 | 0.094 |
| **06H02** | 4 | 4 | 0.25 | 8 | S114G | wt | wt | wt | >32 | 2 | 0.19 | 0.125 |
| **06H06** | 2 | 1 | 0.25 | 4 | wt | wt | wt | wt | 0.75 | 0.5 | 0.094 | 0.094 |
| **06H10** | 4 | 4 | 1 | 8 | S81G | wt | K137N | I460V | >32 | >32 | 3 | 2 |
| **06J34** | 2 | 1 | 0.06 | 4 | wt | wt | wt | I460V | 2 | 0.5 | 0.094 | 0.125 |
| **06J35** | 2 | 2 | 0.25 | 4 | wt | wt | wt | wt | 2 | 0.5 | 0.125 | 0.125 |
| **06J37** | 2 | 1 | 0.25 | 4 | wt | wt | wt | I460V | 2 | 0.5 | 0.125 | 0.125 |
| **06K02** | 2 | 2 | 0.25 | 4 | wt | wt | wt | I460V | 1 | 0.5 | 0.125 | 0.094 |
| **06K08** | 2 | 2 | 0.12 | 4 | wt | wt | K137N | I460V | 0.75 | 0.5 | 0.094 | 0.094 |
| **06K13** | 2 | 1 | 0.25 | 4 | wt | wt | wt | I460V | 2 | 0.75 | 0.125 | 0.125 |
| **06K15** | 2 | 2 | 0.25 | 4 | wt | wt | K137N | I460V | 0.75 | 0.5 | 0.094 | 0.094 |
| **06K19** | 2 | 2 | 0.25 | 4 | wt | D435N | K137N | I460V | 1 | 0.5 | 0.094 | 0.094 |
| **06K23** | 2 | 2 | 0.12 | 4 | wt | wt | K137N | I460V | 0.75 | 0.38 | 0.094 | 0.094 |
| **06L08** | 2 | 1 | 0.25 | 4 | wt | wt | wt | I460V | 1 | 0.75 | 0.125 | 0.125 |
| **06L09** | 2 | 2 | 0.25 | 4 | wt | wt | wt | I460V | 1.5 | 0.75 | 0.125 | 0.125 |
| **06N06** | 2 | 1 | 0.12 | 4 | wt | wt | wt | I460V | 1.5 | 0.5 | 0.125 | 0.125 |
| **06O04** | 2 | 1 | 0.12 | 4 | wt | wt | wt | wt | 1.5 | 0.38 | 0.094 | 0.094 |
| **06O06** | 2 | 1 | 0.12 | 4 | wt | wt | wt | wt | 1.5 | 0.75 | 0.125 | 0.125 |
| **06O09** | 2 | 1 | 0.12 | 4 | wt | wt | K137N | I460V | 0.75 | 0.38 | 0.125 | 0.25 |
| **06O15** | 2 | 2 | 0.12 | 4 | wt | wt | wt | I460V | 2 | 0.75 | 0.125 | 0.125 |
| **06O17** | 2 | 2 | 0.12 | 4 | wt | wt | K137N | I460V | 0.5 | 0.25 | 0.125 | 0.125 |
| **06O19** | 2 | 1 | 0.06 | 4 | wt | wt | wt | I460V | 2 | 0.75 | 0.094 | 0.094 |
| **07A01** | 2 | 1 | 0.25 | 2 | wt | wt | wt | wt | 0.25 | 0.25 | 0.064 | 0.125 |
| **07A40** | 16 | 8 | 1 | 16 | S81F | wt | N49L/K50N/ S79F | wt | >32 | >32 | 3 | 2 |
| **07B16** | 4 | 2 | 0.12 | 4 | wt | wt | wt | I460V | 12 | 0.75 | 0.125 | 0.125 |
| **07C12** | 2 | 1 | 0.25 | 4 | wt | wt | wt | I460V | 1 | 0.75 | 0.125 | 0.125 |
| **07C24** | 2 | 1 | 0.12 | 4 | wt | G434R | K137N | I460V | 0.75 | 0.5 | 0.064 | 0.125 |
| **07F04** | 2 | 1 | 0.12 | 4 | wt | wt | wt | I460V | 1 | 0.75 | 0.125 | 0.125 |
| **07F07** | 4 | 2 | 0.12 | 8 | wt | wt | wt | I460V | 2 | 0.75 | 0.125 | 0.125 |
| **07F08** | 4 | 1 | 0.12 | 8 | wt | wt | wt | I460V | 1 | 0.75 | 0.125 | 0.125 |
| **07G18** | 4 | 1 | 0.12 | 8 | wt | wt | K137N | I460V | 1 | 0.75 | 0.125 | 0.125 |
| **07H01** | 4 | 2 | 0.12 | 8 | wt | wt | S79F | I460V | 4 | 1 | 0.125 | 0.125 |
| **07H04** | 8 | 8 | 2 | 16 | S81F | wt | S79F | I460V | >32 | >32 | 4 | 3 |
| **07I02** | 2 | 1 | 0.12 | 4 | wt | wt | K137N | I460V | 0.75 | 0.75 | 0.125 | 0.064 |
| **07J30** | 16 | 8 | 1 | 16 | S81F | wt | S79F | wt | >32 | >32 | 2 | 2 |
| **07L34** | 4 | 4 | 0.25 | 8 | wt | wt | wt | I460V | 1 | 0.5 | 0.125 | 0.125 |
| **07M27** | 2 | 1 | 0.25 | 4 | wt | wt | R95C | wt | 1 | 0.5 | 0.125 | 0.125 |
| **07O07** | 2 | 1 | 0.12 | 4 | S114G | wt | S79F/N91D | I460V | 16 | 6 | 0.25 | 0.25 |
| **08A02** | 2 | 1 | 0.12 | 4 | wt | wt | wt | wt | 0.5 | 0.25 | 0.125 | 0.064 |
| **08A03** | 2 | 2 | 0.25 | 4 | wt | wt | K137N | I460V | 0.5 | 0.25 | 0.125 | 0.125 |
| **08A12** | 2 | 2 | 0.12 | 4 | wt | wt | wt | I460V | 2 | 0.5 | 0.094 | 0.094 |
| **08A15** | 2 | 2 | 0.25 | 4 | wt | G434R | wt | I460V | 1.5 | 0.75 | 0.125 | 0.5 |
| **08C15** | 2 | 2 | 0.25 | 4 | wt | wt | wt | I460V | 1 | 0.5 | 0.064 | 0.064 |
| **08 E03** | 2 | 2 | 0.25 | 4 | wt | wt | R95C | wt | 0.75 | 0.5 | 0.094 | 0.064 |
| **08 E13** | 2 | 2 | 0.12 | 4 | wt | G434R | wt | I460V | 1.5 | 0.5 | 0.094 | 0.094 |
| **08 E15** | 2 | 2 | 0.25 | 4 | wt | wt | wt | I460V | 6 | 1 | 0.125 | 0.094 |
| **08 E16** | 32 | 16 | 8 | 16 | S81F | wt | S79F | wt | >32 | 12 | 3 | 3 |
| **08G22** | 2 | 1 | 0.12 | 4 | wt | wt | wt | wt | 0.25 | 0.25 | 0.125 | 0.125 |
| **08G25** | 2 | 4 | 0.12 | 4 | wt | G434R | wt | I460V | 2 | 0.5 | 0.125 | 0.094 |
| **08G26** | 2 | 2 | 0.12 | 4 | wt | wt | wt | I460V | 2 | 0.5 | 0.094 | 0.094 |
| **08G28** | 2 | 2 | 0.12 | 4 | wt | wt | wt | I460V | 3 | 0.75 | 0.125 | 0.094 |
| **08I01** | 2 | 2 | 0.25 | 4 | wt | wt | wt | I460V | 0.75 | 0.5 | 0.094 | 0.094 |
| **08J01** | 2 | 2 | 0.25 | 4 | wt | wt | K137N | I460V | 2 | 1 | 0.125 | 0.125 |
| **08J07** | 2 | 2 | 0.25 | 4 | wt | wt | wt | I460V | 0.75 | 0.38 | 0.094 | 0.094 |
| **08J09** | 2 | 2 | 0.25 | 4 | wt | wt | wt | I460V | 2 | 0.5 | 0.125 | 0.094 |
| **08L06** | 2 | 2 | 0.12 | 4 | wt | wt | wt | I460V | 3 | 0.5 | 0.094 | 0.094 |
| **08L13** | 2 | 2 | 0.25 | 4 | wt | wt | wt | wt | 0.75 | 0.5 | 0.125 | 0.125 |
| **08L33** | 16 | 16 | 2 | 16 | S81F | wt | S79F / K137N | I460V | >32 | >32 | 2 | 4 |
| **08L34** | 2 | 2 | 0.25 | 4 | wt | wt | wt | I460V | 1 | 0.5 | 0.125 | 0.5 |
| **08O21** | 2 | 2 | 0.12 | 4 | wt | wt | K137N | I460V | 2 | 0.5 | 0.125 | 0.125 |
| **09B07** | 4 | 2 | 0.25 | 8 | S114G | wt | S79F / N91D | wt | >32 | 4 | 0.125 | 0.125 |
| **09K03** | 2 | 1 | 0.25 | 4 | wt | wt | wt | I460V | 1 | 0.5 | 0.094 | 0.094 |
| **09K10** | 4 | 1 | 0.12 | 4 | wt | wt | wt | I460V | 1 | 0.5 | 0.094 | 0.094 |
| **09K15** | 4 | 2 | 0.12 | 4 | wt | wt | wt | I460V | 4 | 0.5 | 0.094 | 0.094 |
| **09K16** | 2 | 1 | 0.12 | 4 | wt | wt | wt | I460V | 2 | 0.75 | 0.125 | 0.125 |
| **09K22** | 2 | 1 | 0.06 | 4 | wt | wt | S79F | I460V | 3 | 2 | 0.125 | 0.125 |
| **09K32** | 16 | 8 | 1 | 16 | wt | wt | S79F | I460V | >32 | >32 | 2 | 2 |
| **09K33** | 8 | 8 | 0.5 | 16 | wt | wt | S79Y | I460V | >32 | >32 | 2 | 2 |
| **09L20** | 2 | 1 | 0.25 | 4 | wt | wt | wt | I460V | 1 | 0.5 | 0.125 | 0.094 |
| **09L23** | 2 | 2 | 0.25 | 4 | wt | wt | wt | I460V | 0.75 | 0.5 | 0.094 | 0.094 |
| **09L24** | 2 | 1 | 0.25 | 4 | wt | wt | S79F | I460V | 2 | 1.5 | 0.125 | 0.125 |
| **09N30** | 4 | 4 | 0.12 | 8 | wt | wt | wt | I460V | 1.5 | 0.75 | 0.064 | 0.064 |
| **09O15** | 2 | 1 | 0.06 | 4 | wt | wt | wt | I460V | 0.75 | 0.25 | 0.064 | 0.064 |
| **10A09** | 4 | 2 | 0.25 | 4 | wt | wt | wt | I460V | 1.5 | 1 | 0.125 | 0.125 |
| **10A11** | 4 | 2 | 0.5 | 4 | wt | wt | wt | I460V | 1.5 | 1 | 0.19 | 0.19 |
| **10A13** | 2 | 1 | 0.25 | 4 | wt | wt | wt | wt | 0.75 | 0.75 | 0.125 | 0.125 |
| **10A15** | 2 | 1 | 0.25 | 4 | wt | wt | wt | wt | 1 | 0.5 | 0.125 | 0.125 |
| **10A19** | 2 | 1 | 0.12 | 4 | wt | wt | wt | A468T / I493L | 4 | 0.5 | 0.125 | 0.094 |
| **10A23** | 4 | 2 | 0.25 | 8 | wt | wt | wt | wt | 3 | 3 | 0.5 | 0.5 |
| **10A34** | 4 | 2 | 0.25 | 8 | wt | wt | S79F | I460V | 3 | 3 | 0.19 | 0.19 |
| **10D22** | 2 | 2 | 0.25 | 4 | wt | wt | S79I / N91D / E135D | wt | 12 | 6 | 0.25 | 0.25 |
| **10I25** | 2 | 1 | 0.25 | 4 | wt | wt | wt | I460V | 3 | 1 | 0.125 | 0.125 |
| **10K19** | 4 | 4 | 0.12 | 8 | wt | wt | wt | I460V | 6 | 1.5 | 0.125 | 0.125 |
| **10L01** | 2 | 1 | 0.5 | 4 | wt | wt | wt | wt | 0.75 | 0.5 | 0.125 | 0.125 |
| **10L03** | 4 | 4 | 0.25 | 8 | wt | wt | wt | wt | 0.75 | 0.38 | 0.125 | 0.125 |
| **10L16** | 4 | 2 | 0.25 | 8 | wt | wt | wt | I460V | 0.75 | 0.5 | 0.125 | 0.125 |
| **10L18** | 4 | 2 | 0.5 | 4 | wt | wt | wt | I460V | 1.5 | 0.5 | 0.25 | 0.25 |
| **10L36** | 2 | 2 | 0.25 | 4 | wt | wt | wt | I460V | 1.5 | 0.5 | 0.125 | 0.125 |
| **10N11** | 32 | 8 | 4 | 16 | S81F | wt | S79Y | wt | >32 | >32 | 8 | 4 |
| **11A01** | 8 | 4 | 0.25 | 8 | wt | wt | wt | I460V | 4 | 0.5 | 0.125 | 0.125 |
| **11A17** | 8 | 4 | 0.25 | 8 | wt | wt | wt | I460V | 3 | 0.5 | 0.125 | 0.125 |
| **11A21** | 8 | 2 | 0.5 | 4 | wt | wt | wt | I460V | 1.5 | 0.75 | 0.19 | 0.19 |
| **11A23** | 16 | 8 | 1 | 16 | wt | wt | D83N | I460V | >32 | 16 | 1.5 | 1.5 |
| **11A27** | 4 | 2 | 0.25 | 4 | S114G | wt | N91D | I493L | 8 | 1 | 0.19 | 0.125 |
| **11A28** | 8 | 2 | 0.25 | 4 | wt | wt | wt | I460V | 0.75 | 0.5 | 0.125 | 0.125 |
| **11A36** | 16 | 8 | 1 | 16 | wt | wt | D83N | I460V | >32 | 24 | 1 | 1 |
| **11G08** | 4 | 2 | 0.25 | 4 | wt | wt | wt | I460V | 0.75 | 0.5 | 0.125 | 0.125 |
| **11I08** | 4 | 2 | 0.25 | 8 | wt | wt | wt | I460V | >32 | 32 | 1 | 1 |
| **11I10** | 4 | 2 | 0.12 | 8 | wt | wt | wt | I460V | 1 | 0.5 | 0.125 | 0.125 |
| **11I29** | 4 | 2 | 0.12 | 4 | wt | wt | wt | I460V | 1.5 | 0.75 | 0.125 | 0.125 |
| **11I30** | 4 | 2 | 0.12 | 4 | wt | wt | wt | wt | 0.75 | 0.38 | 0.094 | 0.094 |
| **11L12** | 4 | 2 | 0.5 | 8 | wt | wt | wt | I460V | 1 | 0.75 | 0.125 | 0.125 |
| **11O31** | 4 | 2 | 0.25 | 8 | S114G | wt | wt | wt | 4 | 1.5 | 0.125 | 0.125 |
| **12C10** | 16 | 8 | 2 | 8 | S81F | wt | S79F | wt | >32 | >32 | 3 | 3 |
| **12C16** | 4 | 1 | 0.12 | 8 | S114G | wt | wt | I460V | 4 | 3 | 0.5 | 0.5 |
| **12D07** | 4 | 2 | 0.25 | 8 | S114G | wt | N91D | I460V | 3 | 2 | 0.125 | 0.125 |
| **12J05** | 4 | 1 | 0.25 | 4 | wt | wt | wt | I460V | 1.5 | 1 | 0.125 | 0.125 |
| **12J12** | 4 | 2 | 0.25 | 4 | wt | wt | wt | I460V | 1 | 1 | 0.094 | 0.094 |
| **12K21** | 4 | 2 | 0.5 | 4 | wt | wt | wt | I460V | 1 | 1 | 0.125 | 0.125 |
| **12K22** | 4 | 2 | 0.25 | 4 | wt | wt | wt | I460V | 1 | 0.75 | 0.064 | 0.064 |
| **12K23** | 4 | 1 | 0.12 | 4 | wt | wt | wt | I460V | 1 | 0.75 | 0.125 | 0.094 |
| **12K25** | 4 | 2 | 0.25 | 4 | wt | wt | wt | I460V | 1 | 1 | 0.125 | 0.125 |
| **12K32** | 4 | 4 | 0.12 | 4 | wt | wt | wt | I460V | 0.5 | 0.5 | 0.125 | 0.125 |
| **12L01** | 4 | 4 | 0.25 | 4 | wt | wt | wt | I460V | 1 | 1 | 0.125 | 0.125 |
| **12L03** | 4 | 2 | 0.12 | 4 | wt | wt | wt | I460V | 1 | 0.75 | 0.094 | 0.094 |
| **12L10** | 4 | 4 | 0.12 | 4 | wt | wt | wt | I460V | 1 | 0.75 | 0.125 | 0.125 |
| **12L13** | 4 | 4 | 0.5 | 4 | wt | wt | wt | I460V | 1 | 0.75 | 0.125 | 0.125 |
| **12L14** | 4 | 4 | 0.12 | 4 | wt | wt | wt | wt | 0.25 | 0.25 | 0.094 | 0.094 |
| **12L18** | 4 | 4 | 0.5 | 4 | wt | wt | wt | I460V | 1 | 0.75 | 0.094 | 0.094 |
| **12L20** | 4 | 2 | 0.12 | 2 | wt | wt | wt | wt | 0.75 | 0.5 | 0.094 | 0.094 |
| **12L36** | 4 | 2 | 0.12 | 4 | wt | wt | wt | I460V | 1 | 0.75 | 0.125 | 0.125 |
| **12L37** | 4 | 2 | 0.12 | 4 | wt | wt | wt | I460V | 1 | 1 | 0.094 | 0.094 |
| **12L38** | 4 | 2 | 0.06 | 2 | wt | wt | wt | I460V | 1.5 | 1 | 0.094 | 0.094 |
| **12M02** | 4 | 4 | 0.25 | 4 | wt | wt | S79F / N91D | I460V | 4 | 1 | 0.125 | 0.125 |
| **12M03** | 4 | 2 | 0.25 | 4 | wt | wt | wt | I460V | 1.5 | 1 | 0.125 | 0.125 |
| **12M06** | 4 | 4 | 0.12 | 4 | wt | wt | wt | I460V | 0.75 | 0.75 | 0.064 | 0.064 |
| **12M09** | 8 | 4 | 0.12 | 8 | wt | wt | wt | I460V | 1 | 0.75 | 0.125 | 0.094 |
| **12N02** | 4 | 2 | 0.25 | 4 | S114G | wt | wt | I460V | 3 | 1.5 | 0.25 | 0.19 |
| **12O33** | 4 | 2 | 0.12 | 4 | wt | wt | wt | I460V | 1 | 0.75 | 0.125 | 0.125 |
| **12O34** | 4 | 2 | 0.12 | 4 | wt | wt | wt | I460V | 1 | 1 | 0.125 | 0.125 |
| **12O40** | 8 | 4 | 0.25 | 8 | wt | wt | wt | wt | 2 | 1 | 0.125 | 0.125 |
| **13A12** | 4 | 2 | 0.12 | 8 | wt | wt | wt | I460V | 1.5 | 0.5 | 0.125 | 0.125 |
| **13A21** | 4 | 2 | 0.25 | 8 | wt | wt | wt | I460V | 3 | 1 | 0.19 | 0.19 |
| **13A22** | 8 | 2 | 0.25 | 8 | wt | wt | wt | I460V | 4 | 1.5 | 0.125 | 0.125 |
| **13A26** | 4 | 2 | 0.25 | 4 | wt | wt | wt | I460V | 2 | 1 | 0.125 | 0.125 |
| **13A30** | 4 | 2 | 0.25 | 4 | wt | wt | wt | I460V | 2 | 1.5 | 0.125 | 0.125 |
| **13A35** | 4 | 2 | 0.06 | 8 | wt | wt | D83N | I460V | 4 | 2 | 0.125 | 0.125 |
| **13B01** | 8 | 2 | 0.5 | 8 | wt | wt | wt | I460V | 3 | 0.75 | 0.19 | 0.19 |
| **13B02** | 4 | 2 | 0.25 | 4 | wt | wt | wt | I460V | 1 | 0.75 | 0.125 | 0.125 |
| **13B06** | 4 | 2 | 0.25 | 4 | wt | wt | wt | I460V | 1 | 0.5 | 0.125 | 0.125 |
| **13B08** | 4 | 2 | 0.25 | 8 | wt | wt | wt | wt | 2 | 1 | 0.125 | 0.125 |
| **13B09** | 4 | 2 | 0.12 | 4 | wt | wt | wt | I460V | 1.5 | 0.5 | 0.094 | 0.094 |
| **13C24** | 4 | 2 | 0.12 | 8 | wt | wt | wt | I460V | 8 | 4 | 0.19 | 0.19 |
| **13C28** | 8 | 4 | 2 | 8 | S81F | wt | wt | I460V | >32 | >32 | 4 | 4 |
| **13D16** | 4 | 2 | 0.12 | 4 | wt | wt | wt | I460V | 2 | 1 | 0.19 | 0.125 |
| **13E07** | 4 | 2 | 0.25 | 4 | wt | wt | wt | I460V | 2 | 1 | 0.125 | 0.125 |
| **13E24** | 4 | 4 | 0.12 | 4 | S114G | wt | wt | I460V | 1.5 | 0.5 | 0.125 | 0.125 |
| **13F15** | 4 | 2 | 0.12 | 8 | S114G | wt | wt | wt | 8 | 1 | 0.19 | 0.19 |
| **13G08** | 4 | 1 | 0.06 | 8 | S114G | wt | N91D / E135D | wt | 16 | 1 | 0.19 | 0.19 |
| **13J24** | 32 | 16 | 2 | 32 | S81F | wt | wt | I460V | >32 | >32 | 4 | 4 |
| **13J29** | 4 | 2 | 0.12 | 8 | wt | wt | wt | I460V | 1.5 | 0.75 | 0.125 | 0.125 |
| **13K02** | 4 | 1 | 0.25 | 8 | wt | wt | wt | I460V | 2 | 1 | 0.19 | 0.19 |
| **13K03** | 4 | 2 | 0.12 | 8 | wt | wt | wt | I460V | 1 | 0.75 | 0.125 | 0.125 |
| **13K06** | 4 | 1 | 0.12 | 8 | wt | wt | wt | I460V | 1 | 0.5 | 0.125 | 0.125 |
| **13K07** | 4 | 0.5 | 0.12 | 8 | wt | wt | K137N | I460V | 1.5 | 0.75 | 0.125 | 0.125 |
| **13K13** | 4 | 2 | 0.12 | 8 | wt | wt | K137N | I460V | 2 | 1 | 0.125 | 0.125 |
| **13K14** | 4 | 2 | 0.12 | 4 | wt | wt | wt | I460V | 1.5 | 1 | 0.125 | 0.125 |
| **13K18** | 4 | 2 | 0.25 | 8 | wt | wt | wt | wt | 1.5 | 1 | 0.19 | 0.19 |
| **13K19** | 8 | 4 | 0.25 | 8 | wt | wt | S79F | I460V | 6 | 3 | 0.25 | 0.25 |
| **13L03** | 4 | 2 | 0.12 | 8 | wt | wt | wt | I460V | 1.5 | 0.75 | 0.125 | 0.125 |
| **13L04** | 16 | 8 | 2 | 16 | S81Y | wt | S79F | I460V | 24 | 24 | 4 | 4 |
| **13L14** | 4 | 1 | 0.12 | 8 | wt | wt | wt | I460V | 3 | 1 | 0.19 | 0.19 |
| **13L15** | 4 | 2 | 0.12 | 8 | wt | wt | wt | I460V | 1.5 | 0.75 | 0.125 | 0.125 |
| **13L23** | 4 | 2 | 0.12 | 8 | wt | wt | wt | I460V | 3 | 1 | 0.19 | 0.19 |
